# Associations between Heart Rate Variability and Brain Activity during a Working Memory Task: A Preliminary Electroencephalogram Study on Depression and Anxiety Disorder

**DOI:** 10.3390/brainsci12020172

**Published:** 2022-01-28

**Authors:** Deokjong Lee, Woohyun Kwon, Jaeseok Heo, Jin Young Park

**Affiliations:** 1 Department of Psychiatry, Yongin Severance Hospital, Yonsei University College of Medicine, Gyeonggi-do 16995, Korea; pangelt@yuhs.ac; 2 Institute of Behavioral Science in Medicine, Yonsei University College of Medicine, Seoul 03722, Korea; kwonwoohyun@yuhs.ac (W.K.); heojs@yuhs.ac (J.H.); 3 Graduate Program in Cognitive Science, The Graduate School of Yonsei University, Seoul 03722, Korea; 4 Department of Laboratory Medicine, Yongin Severance Hospital, Yonsei University Health System, Gyeonggi-do 03722, Korea

**Keywords:** electroencephalogram, heart rate variability, working memory

## Abstract

Heart rate variability (HRV) has been suggested to reflect executive function and related neural activity. Executive dysfunction has been suggested to play an important role in the pathophysiology of emotional disorders. The purpose of this study was to investigate whether HRV showed a significant correlation with electroencephalogram (EEG) during a working memory performance in patients with depressive or anxiety disorder. A retrospective analysis was conducted with data from 61 patients with depressive disorder (43 women and 18 men) and 59 patients with anxiety disorder (35 women and 24 men). HRV was measured in the resting state, and EEG was recorded in the resting state and during the execution of a working memory task. It was performed in patients with depressive and anxiety disorder, and the paired sample t-test between resting state and task performance, as well as the partial correlation analysis between HRV and EEG, was conducted. Both depressed and anxious patients showed weaker beta relative power during the working memory task compared to the rest period. The resting-state EEG did not correlate with HRV parameters in both groups. In depressed patients, HRV showed a positive correlation with delta power during the task and a negative correlation with beta relative power during the task. In patients with anxiety disorder, HRV showed a significant positive correlation with theta power of the right frontal region during the task. Our results suggest that HRV would be related to executive-function-related neural activity in patients with depressive or anxiety disorder. Future studies with more subjects, including healthy controls, are needed to verify the correlation between HRV and EEG and to come up with a more comprehensive picture of neurobiological changes in emotional disorders.

## 1. Introduction

Periodic changes in heart rate over time are referred to as heart rate variability (HRV), which is known to mainly reflect the function of the autonomic nervous system (ANS) [1]. HRV is known to be closely related to psychological conditions such as depression, anxiety, and stress and has been used as a useful bio-signal indicator to reflect mental health status [2,3]. Numerous studies have suggested that HRV tends to be significantly lower in patients with several psychiatric disorders, including major depression, bipolar disorder, panic disorder, and posttraumatic stress disorder [4,5,6,7]. On the other hand, HRV is also suggested to reflect executive control functions such as the inhibition of unwanted responses and the updating and monitoring of working memory representations [8]. Several behavioral task studies have established that higher resting HRV is associated with more adaptive executive control over stimuli [9].

The central autonomic network is the functionally connected brain region responsible for reflexive and modulatory control of ANS functioning [10]. The central autonomic network includes several brain regions of cortical functional networks such as the cingulate cortex, the insula, and the medial prefrontal cortex [11]. Since HRV is a bio-signal that reflects ANS functioning, numerous studies have used HRV to explore the neural activity of the central autonomic network [12]. These studies have suggested that the brain regions of the task-positive networks are involved in the sympathetic regulation, whereas the core areas of the default mode network show parasympathetic predominance [13]. However, most of the previous studies have been based on neuroimaging. Functional neuroimaging has the advantage of remarkable spatial resolution, but the confounding effects also exist, as it indirectly reflects the neural activity through the BOLD signals [14]. Neurophysiological indicators such as electroencephalogram (EEG) can more directly reflect brain activity and have an excellent temporal resolution. The investigations of the correlations between HRV and neurophysiological features are relatively scarce, and these efforts may contribute to elucidating neural correlates related to ANS regulation.

Emotional disturbances such as depression and anxiety disorder are known to accompany changes in brain activity and ANS dysfunction [15]. HRV and EEG measurements during a short resting period are widely used in clinical settings as bio-signals reflecting the neurobiological changes in emotional disorders [16]. This study retrospectively analyzed the relationship between quantitative EEG data and resting HRV of patients who firstly visited a psychiatric clinic with emotional distress. This study involved two types of emotional disturbances (depressive disorder and anxiety disorder) and explored HRV-correlated EEG features that were shared or distinct between the two groups. The purpose of this study was to investigate whether the brain activity of neural networks involved in ANS regulation would be significantly reflected through EEG indicators. We also speculated that the significant correlation between HRV and EEG, measured independently of each other, could support the reliability of these two bio-signals in reflecting neurobiological changes in emotional disorders.

In particular, we acquired EEG data not only in the resting state but also during the working memory task. It has been suggested that executive functions, including working memory, [17] play a crucial role in the pathophysiology of emotional disorders [18,19]. Alterations in the prefrontal circuit responsible for executive function are suggested to weaken the emotional regulatory capacity and thus predispose patients to emotional disorders [20]. Previous evidence regarding HRV has suggested that there is an association between HRV and executive control because HRV is linked to the functioning of the prefrontal-subcortical inhibitory circuits [21]. Therefore, EEG markers during executive function, which are correlated with HRV, are speculated to reflect neurobiological changes in emotional disorders.

## 2. Methods

### 2.1. Subjects

This study retrospectively analyzed EEG and HRV data of subjects who visited a psychiatric outpatient clinic for anxiety or depression. Adult male and female patients who visited the outpatient clinic from March to December 2020 were included. During the study period, 157 subjects completed both EEG and HRV. They were assessed for a psychiatric diagnosis according to the Diagnostic and Statistical Manual of Mental Disorders (DSM-5) criteria through the Mini International Neuropsychiatric Interview (MINI) [22]. Among them, those diagnosed with depressive disorders or anxiety disorders in the DSM-5 were included in this study. In total, there were 120 subjects with 42 male and 78 female adults. The full-scale intelligence quotient (FSIQ) of each subject was measured through the Korean version of Wechsler adult intelligence scale IV (K-WAIS-IV) [23]. Depression and anxiety levels of the subjects were assessed by the hospital anxiety and depression scale (HADS) [24].

### 2.2. HRV Test

HRV values were measured using the stress-measuring equipment, SA-3000P (medicine Co., Ltd., Seoul, Korea). In a stable state in which the subject sat comfortably, electrodes were attached to the left and right wrists and left ankles, respectively, and then, the electrocardiogram (ECG) was measured for 5 min. HRV was calculated through R-R variability in the ECG data, and frequency domain analysis was performed using Lead II, where the R-peak was most evident. The digital band-stop filter (notch filter) was used to reduce the influence of confounding signals such as noise caused by muscle tension [25]. After filtering, the mixed high-frequency and low-frequency components were separated by performing a Fast Fourier Transform (FFT). HF power was calculated as the area occupied by the frequency range of 0.15–0.4 Hz [26]. Low frequency (LF) power was calculated as the area occupied by the frequency range of 0.04–0.15 Hz.

### 2.3. EEG Data Recording

**Figure 1:**
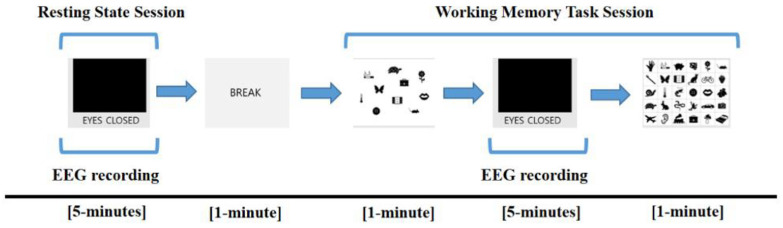
Electroencephalogram (EEG) recording protocol. EEG was measured during the resting session for 5 min and retention interval period for 5 min in the working memory task session.

The overall protocol for measuring EEG data is described in Figure 1. Subjects sat comfortably in a chair, and EEG signals were measured for 5 min with eyes closed (resting-state session). During the resting-state session, subjects were instructed to remain awake but relaxed without engaging in any particular thought. After a break time of 1 min, subjects performed a working memory task for a total of 7 min (working memory task session). The task session proceeded in the following order: (1) subjects were presented with a paper on which 10 target items were drawn and were instructed to memorize the items for 1 min; (2) subjects were asked to close their eyes again and were instructed to retain the target items in mind for 5 min. During this 5 min, EEG signals were acquired; (3) subjects were presented with a piece of paper with 30 items for 1 min and were instructed to select the items corresponding to the 10 target items they had been instructed to retain in mind.

EEG signals were recorded via the Net station version 5.4 software (Electrical Geodesics, Eugene, OR, USA) and a 64-channel HydroCel Geodesic Sensor Net (Electrical Geodesics Inc., Eugene, OR, USA) based on the modified international 10–20 system (Applied Neuroscience, St. Peterburg, FL, USA). The sponge-based carbon fiber electrodes (Ag/AgCl-coated, carbon-filled plastic electrodes with a sponge) were used and referenced to the vertex electrode (Cz) of the scalp. The EEG data were resampled at 1 kHz using the Geodesic EEG system 400 (Electrical Geodesics, Inc.) and filtered with a bandpass filter set at 0.1–100 Hz and notch filter set at 60 Hz.

### 2.4. EEG Data Preprocessing and Power Spectra Analysis

EEG data were pre-processed and analyzed using the MATLAB 2016b (The MathWorks, Natick, MA, USA) and the EEGLAB toolbox [27]. The Harvard Automated Processing Pipeline for EEG (HAPPE) was applied [28]. In the bad channel rejection processing step, bad channels whose probability fell more than 3 standard deviations (SDs) from the mean are eliminated. Removed channels were spherically interpolated with Legendre polynomials up to the 7th order of their signal. The independent component analysis (ICA) using the multiple artifact rejection algorithm (MARA) was conducted to remove components with artifact probability of greater than 0.8 [29]. 

In power spectra analysis, five types of frequency bands were defined: delta (from 1 to 4 Hz), theta (from 4 to 8 Hz), alpha (from 8 to 13 Hz), beta (from 13 to 30 Hz), and gamma (from 30 to 50 Hz). Five components were separated for each subject by performing a Fast Fourier Transform (FFT). Time windows of 4000 ms with an 8 ms overlap and the Hamming window were applied. The outliers that were far from the spectral value distribution of each frequency band at the 0.05 significance level were eliminated. The absolute powers were averaged over all of the time windows and frequency bands. The relative powers were recorded by calculating absolute power in each frequency band as a percentage of the absolute power summed over the five frequency bands. The topographical distribution plots showed the average absolute and relative power of frequency bands acquired from the grand average across subjects during the resting state session and the working memory task session.

### 2.5. Statistical Analyses

Quantified HRV and EEG values were used for statistical analyses. Analyses were performed independently for each group of subjects with depressive and anxiety disorders. Statistical analyses were performed using SPSS statistical software for Windows, version 25.0 (SPSS, Chicago, IL, USA) and MATLAB version R2020b statistical toolbox (The MathWorks, Inc., Natick, MA, USA). The thresholds for statistical significance were set to *p* < 0.05. For EEG analyses and topographic plots formation, the False Discovery Rate (FDR) multiple comparison corrections were applied [30]. The paired sample *t*-tests were applied to compare the resting-state session and the working memory task session. The partial correlation analyses were conducted to explore the EEG indices correlated with HRV. Age, sex, FSIQ, and HADS scores were entered into correlation analyses as covariates.

## 3. Results

### 3.1. Demographic and Clinical Characteristics of Subjects

Table 1 shows the demographic and clinical characteristics of the subjects. Group 1 included 61 subjects with depressive disorder, of which 18 were male and 43 were female. Group 2 included 59 subjects with anxiety disorder, of which 24 were male and 35 were female. The detailed diagnoses of each of the 61 depressed subjects were as follows: 24 major depressive disorder, 16 unspecified depressive disorder, 11 dysthymia, 9 adjustment disorders with depressed mood, and 1 cyclothymic disorder. The detailed diagnoses of each of the 59 subjects with anxiety disorder were as follows: 31 panic disorder, 13 generalized anxiety disorder, 5 adjustment disorder with anxiety, 4 social anxiety disorder, 4 unspecified anxiety disorder, and 2 specific phobias. Subjects with depressive disorder scored significantly higher on the depression subscale of the HADS compared with subjects with anxiety disorder (*p* < 0.001). Other variables were not significantly different between groups.

### 3.2. Comparison between Rest Period and Working Memory Task Performance

**Figure 2:**
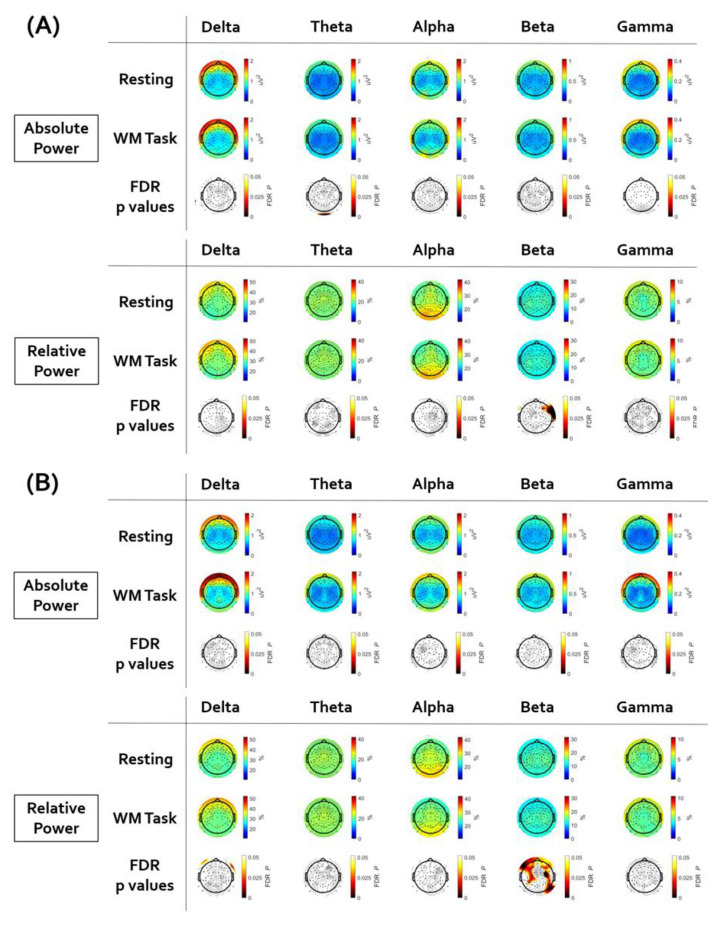
The results of the paired sample t-test between resting state and working memory task. Electroencephalogram (EEG) topographic map after false discovery rate (FDR) correction. (**A**) In subjects with depressive disorder, the beta relative power of frontal electrodes was weaker during the working memory task performance than during the rest period. (**B**) In subjects with anxiety disorder, the beta relative power of wide ranges of regions was weaker during the working memory task performance than during the rest period.

Figure 2 illustrates the topographical distribution plots for the comparison between the resting-state session and the working memory task session. Statistical values of all paired *t*-tests performed are presented in Supplementary Tables S1–S5. In subjects with depressive disorder, the beta relative powers of frontal electrodes (FT8, F8, F6, and F4) were weaker during the working memory task performance than during the rest period. In subjects with anxiety disorder, the beta relative powers of a wide range of regions (AF4, F2, Fp2, F1, Fp1, AF3, F3, F5, FC5, FC3, F7, FT7, FC4, FC6, F8, F6, F4, C1, C3, CP1, CP5, CP6, C6, C4, T6, TP8, P5, P3, P10, P6, and A2) were weaker during the working memory task performance than during the rest period.

### 3.3. Analysis of Correlation between EEG Signal and LF-HRV

**Figure 3:**
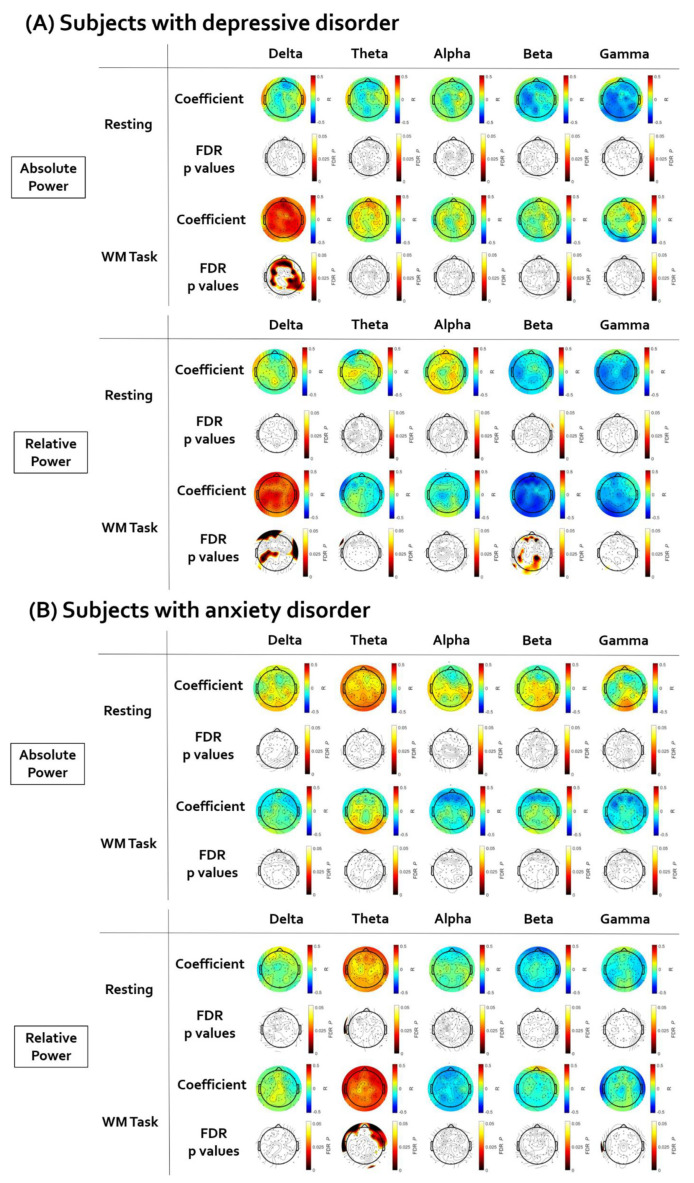
The results of the partial correlation analyses between resting low-frequency heart rate variability (LF-HRV) and electroencephalogram (EEG). The EEG topographic map after false discovery rate (FDR) correction. (**A**) In subjects with depressive disorder, the LF-HRV was positively correlated with delta power and negatively correlated with beta relative power. (**B**) In subjects with anxiety disorder, the LF-HRV was positively correlated with theta relative power.

Figure 3 illustrates the topographical distribution plots for the EEG findings showing significant correlations with LF-HRV. Statistical values of all correlation analyses performed are presented in Supplementary Tables S6–S15.

First, in subjects with depressive disorder, the brain regions showing a significant correlation between resting LF-HRV and EEG power were investigated. There was no significant correlation between resting LF-HRV and EEG power in the resting state. The positive correlation between resting LF-HRV and delta absolute power during working memory task was significant in wide ranges of regions (coefficient value: from 0.336 to 0.502; AF4, F2, Fp2, Fz, AFz, F1, Fp1, AF3, F3, F5, FC5, FC3, FT7, FC4, FC6, F4, C3, C5, CP5, CP2, CP6, C6, P5, P3, P1, PO3, PO4, P2, P4, P6, T6, TP7, TP8, A2). The delta relative power was also positively correlated with LF-HRV in several electrodes (coefficient value: from 0.336 to 0.432; AFz, Fp1, C1, C3, CP1, C5, CP5, C6, Cz, TP7, T4, P5, P3, P9). The negative correlation between resting LF-HRV and beta relative power during working memory task was significant in several electrodes (coefficient value: from 0.336 to 0.432; AF4, F2, AFz, F1, AF3, C3, C5, CP5, TP7, P5, P3, POz, PO4, P2, P4, A1, O1, Oz, O2). 

Second, in subjects with anxiety disorder, the brain regions showing a significant correlation between resting LF-HRV and EEG power were investigated. There was no significant correlation between resting LF-HRV and EEG power in the resting state. The positive correlation between resting LF-HRV and theta relative power during the working memory task was significant in several frontal electrodes (coefficient value: from 0.376 to 0.445; AF4, Fp2, F3, FT8, FC6, F8, F6, F4) and the T4 electrode.

### 3.4. Analysis of Correlation between EEG Signal and HF-HRV

**Figure 4:**
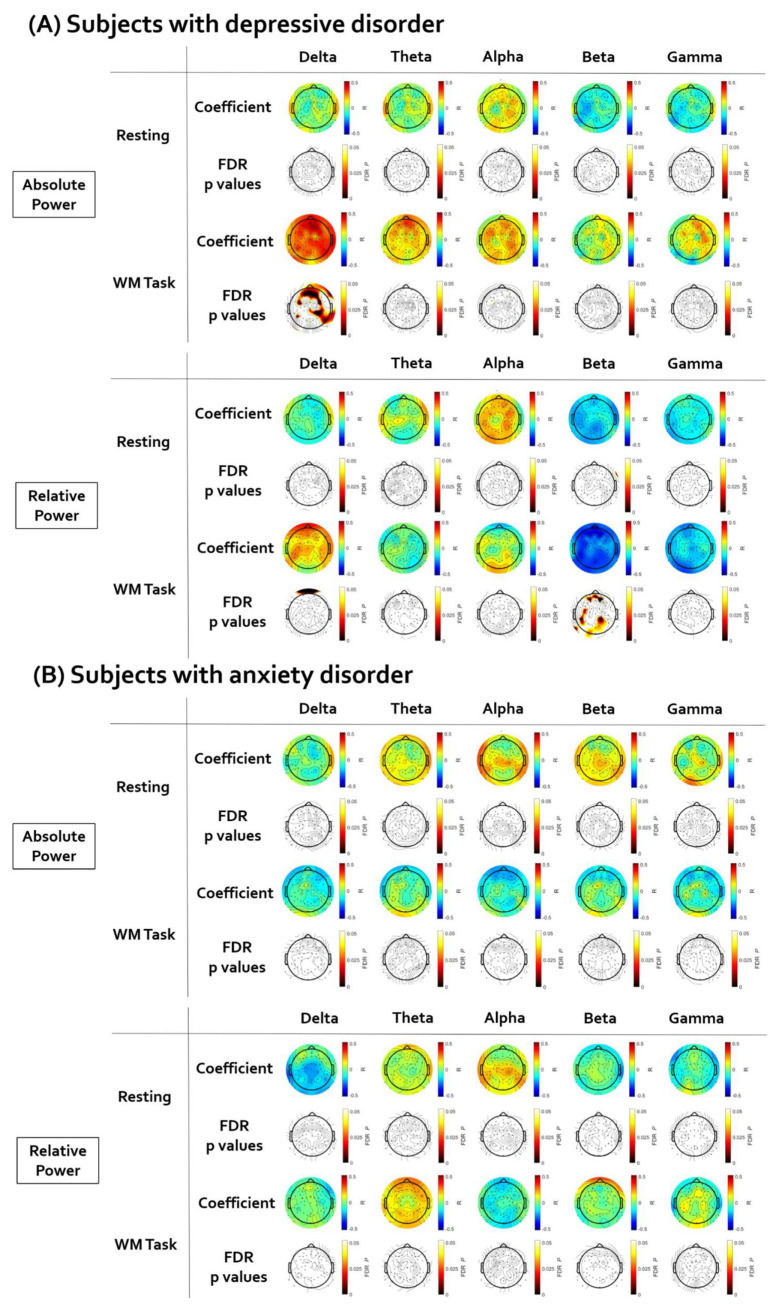
The results of the partial correlation analyses between resting high-frequency heart rate variability (HF-HRV) and electroencephalogram (EEG). The EEG topographic map after false discovery rate (FDR) correction. (**A**) In subjects with depressive disorder, the HF-HRV was positively correlated with delta absolute power and negatively correlated with beta relative power. (**B**) There was no significant correlation between HF-HRV and EEG in subjects with anxiety disorder.

Figure 4 illustrates the topographical distribution plots for the EEG findings showing significant correlations with LF-HRV. Statistical values of all correlation analyses performed are presented in Supplementary Tables S16–S25. First, in subjects with depressive disorder, the brain regions showing a significant correlation between resting HF-HRV and EEG power were investigated. There was no significant correlation between resting HF-HRV and EEG power in the resting state. The positive correlation between resting HF-HRV and delta absolute power during working memory task was significant in a wide range of regions (coefficient value: from 0.319 to 0.487; AF4, F2, FCz, Fp2, Fz, AFz, F1, Fp1, AF3, F3, F5, FC5, FC3, FC4, FC6, F8, F4, P5, P3, PO3, P4, P6, CP2, CP6, C6, Cz, T6, TP8, T4, A2). The negative correlation between resting HF-HRV and beta relative power during working memory task was significant in several electrodes (coefficient value: from −0.313 to −0.460; AF4, F2, AFz, F1, AF3, C3, C5, CP5, TP7, P5, P3, POz, PO4, P2, P4, A1, O1). Second, in subjects with anxiety disorder, the brain regions showing a significant correlation between resting HF-HRV and EEG power were investigated. There was no significant correlation between resting HF-HRV and EEG power in resting state and working memory task performance.

## 4. Discussion

This study analyzed the correlation between HRV and EEG data in patients with depressive and anxiety disorders. In both depressed and anxious patients, resting-state EEG did not correlate with HRV parameters. However, HRV showed a significant positive correlation with EEG during the working memory task in several waveforms. These findings are consistent with the prediction that HRV would be more significantly related to EEG features during the working memory task. In this study, a significant correlation with EEG during the working memory task was found not only in HF-HRV but also in LF-HRV. Previous research on HRV has mainly focused on the relationship between HF-HRV and executive function [31]. Because the prefrontal-subcortical inhibitory circuits involve inhibitory inputs to the heart via the vagus nerve, executive function is closely related to vagally mediated HRV parameters [32]. In general, it has been considered that HF-HRV is more prominently dominated by the parasympathetic tone than LF-HRV [33]. However, some evidence has suggested that LF-HRV is not only affected by sympathetic flow but also parasympathetic tone [34]. Several studies have also indicated that LF-HRV is related to executive function, including working memory [35,36]. We suggest that the results of this study support that HRV parameters would reflect brain activity involved in executive functioning, including working memory utilization.

The beta relative power was the frequency band in which a significant difference between the rest period and working memory task was found. The decrease in the beta wave during working memory load was consistent with previous EEG studies [37,38]. Recent studies also suggested that the change in beta wave plays a major role as a gatekeeper in retaining information in the working memory [39]. In correlation analysis, HRV of depressed patients showed a significant negative correlation with beta relative power during the working memory task. Taken together, our findings suggest that HRV could reflect brain activity related to working memory capacity in depression, and the relative power of beta waves would index this relationship.

Subjects with depressed mood showed a significant positive correlation between HRV and delta power during the working memory task. Overall, except for sleep state [40], in people with depression, it has been suggested that the delta power abnormally increases along with the decrease in vigilance, [41] and the decrease in the delta wave was related to the improvement of depression [42]. Considering the tendency of low HRV in depression, it was somewhat unexpected that a decrease in HRV was correlated with a decrease in delta power in this study. We suggest that the context in which delta power was acquired should be considered. We measured EEG during the internal concentration without engagement to external stimuli, and a previous study showed that delta power increases during this cognitive process in normal adults [43]. We interpret that low HRV in depressed patients may be related to the inefficiency of brain activation involved with internal concentration.

Subjects with anxiety disorder showed a significant positive correlation between LF-HRV and theta relative power during the working memory task. The brain areas that showed this significant correlation corresponded to the right frontal regions. Theta wave is known to be activated by relaxed attention [44] and has been used in biofeedback treatment for anxiety [45]. In particular, the theta band in the right frontal region during the cognitive task has been recently proposed as a biomarker for anxiety disorder [46,47]. HRV has been used as a neurobiological marker reflecting ANS dysfunction in anxiety disorders [15]. The correlation between LF-HRV and right frontal theta power supports that these bio-signal indicators could reflect neurobiological changes in anxiety disorder. However, the HF component of HRV has been suggested to be a more reliable indicator for anxiety disorders [48]. Therefore, it is necessary to reconsider the implications of our findings through further studies.

This study has several important limitations to be acknowledged. First, this study included broad spectrums of depressive and anxiety disorders. We tried to explore HRV-EEG as a trans-diagnostic feature [49] and entered severity scales of depression and anxiety as covariates. However, in order to validate the results of this study, a further study with a larger size of sample including a more focused and refined group of subjects would be needed. Second, it would be essential to investigate the correlation between HRV and EEG data in a normal control group in order to interpret the correlated features as being associated with psychiatric abnormalities. Since this study was a retrospective analysis of patients who visited the hospital, it was impossible to establish a control group. A prospective study including a control group based on this preliminary study is needed to elaborate on our current findings. We expect to derive more sophisticated results by applying a statistical methodology that can analyze between-group differences in future studies. Third, only HRV in the resting state was analyzed in this study. Like EEG, HRV can provide meaningful implications not only in the resting state but also in response to cognitive loads. Including HRV reactivity to working memory tasks in the analysis may provide a more holistic picture of the relationship between HRV and EEG. Fourth, we used only EEG acquired from the retaining memory period for analysis. This was to target the period most similar to the resting state (the period with eyes closed) but with only the cognitive load added. However, due to the limited measurement range, only some aspects of working memory could be reflected. Fifth, this study conducted only the correlational analysis between HRV and EEG but did not include the analysis of causality between the two bio-signals. Therefore, there is a limit to suggesting the role of HRV and EEG in the pathophysiological model of depression and anxiety.

Although this study has many limitations as a preliminary study, we believe that the results of this study can provide some important implications of HRV and EEG on the neurobiological basis of emotional disturbance. In particular, only EEG signals measured during working memory performance, not during rest periods, correlated significantly with HRV. Our results suggest that in depressed and anxious patients, HRV is associated with the activity of brain neural networks involved in cognitive processing. We suggest that HRV and EEG, which showed a significant correlation with each other, could reflect neurobiological changes in emotional disorders.

## Figures and Tables

**Table 1:** Demographic and clinical characteristics of study participants.

	Group 1 (Depressive Disorders, *N* = 61)		Group 2 (Anxiety Disorder, *N* = 59)			
	Mean	SD	Mean	SD	*t*	*p*
Age (years)	52.2	18.9	46.6	18.0	1.682	0.950
FSIQ	99.9	9.2	98.5	10.6	0.721	0.472
HADS—Depression	13.8	4.6	10.5	4.4	3.963	<0.001
HADS—Anxiety	11.1	4.8	11.5	4.3	−0.512	0.610
LF-HRV (ln)	4.6	1.5	5.0	1.2	−1.479	0.142
HF-HRV (ln)	4.5	1.5	4.7	1.3	−0.820	0.414
Working memory task						
Number of items memorized correctly	8.8	1.9	9.3	1.0	−1.523	0.130

## Data Availability

All data analyzed during this study are included in the published article and are available from the corresponding author on reasonable request.

## Supplementary Materials

The following are available online at https://www.mdpi.com/article/10.3390/brainsci12020172/s1, Tables S1–S5. Statistical values of the paired sample *t*-test; Tables S6–S15. Statistical values of the partial correlation analyses for LF-HRV; Tables S16–S25. Statistical values of the partial correlation analyses for HF-HRV
