# Supplementary material for: Associations between Heart Rate Variability and Brain Activity during a Working Memory Task: A Preliminary Electroencephalogram Study on Depression and Anxiety Disorder
Source: Brain Sci. 2022 Jan 28;12(2):172. doi: 10.3390/brainsci12020172 (PMC8870686; doi:10.3390/brainsci12020172)
Supplement: Supplementary file 1 [file brainsci-12-00172-s001.zip › brainsci-1530820-supplementary.pdf]

Supplementary Table S1. Paired t-test results for comparison between rest period and working memory performance (delta frequency band).

		Group 1. Depressive disorder				Group 2. Anxiety disorder			
Electrodes		Absolute		Relative		Absolute		Relative	
No.	Name	t	p (FDR)	t	p (FDR)	t	p (FDR)	t	p (FDR)
01	AF4	0.511	0.741	-1.567	0.404	-1.555	0.190	-1.815	0.128
02	F2	0.189	0.932	-1.158	0.408	-1.969	0.190	-1.895	0.119
03	FCz	-1.886	0.316	-1.087	0.418	-1.949	0.190	-0.985	0.374
04	Fp2	-0.259	0.889	-1.713	0.404	-1.393	0.218	-1.531	0.196
05	Fz	0.005	0.995	-0.895	0.454	-1.321	0.232	-1.289	0.250
06	FC1	-0.643	0.709	-1.185	0.404	-1.538	0.190	-2.302	0.085
07	AFz	-0.124	0.951	-1.409	0.404	-1.606	0.190	-1.439	0.227
08	F1	-0.293	0.877	-1.017	0.431	-2.644	0.190	-2.361	0.085
09	Fp1	-0.090	0.962	-0.651	0.578	-2.244	0.190	-2.112	0.092
10	AF3	-0.066	0.963	-1.284	0.404	-1.560	0.190	-2.446	0.085
11	F3	0.145	0.951	-1.676	0.404	-1.096	0.303	-1.854	0.126
12	F5	-1.139	0.536	-2.003	0.404	-1.860	0.190	-1.911	0.119
13	FC5	-0.859	0.623	-1.203	0.404	-1.899	0.190	-2.346	0.085
14	FC3	-1.057	0.578	-1.123	0.418	-1.942	0.190	-2.666	0.078
15	C1	-0.727	0.704	-1.591	0.404	-1.866	0.190	-2.657	0.078
16	F7	-1.552	0.380	-2.066	0.404	-1.589	0.190	-2.153	0.092
17	FT7	-1.919	0.316	-1.710	0.404	-1.058	0.316	-1.582	0.188
18	C3	-0.919	0.623	-1.246	0.404	-2.173	0.190	-3.002	0.078
19	CP1	-0.562	0.741	-1.679	0.404	-1.516	0.190	-2.210	0.092
20	C5	-1.398	0.432	-1.504	0.404	-1.779	0.190	-2.094	0.092
21	T3	-1.918	0.316	-1.204	0.404	-1.343	0.228	-2.644	0.078
22	TP7	-1.868	0.316	-1.661	0.404	-1.979	0.190	-2.140	0.092
23	CP5	-1.270	0.476	-1.684	0.404	-2.056	0.190	-2.157	0.092
24	P5	-1.432	0.426	-1.363	0.404	-2.243	0.190	-2.258	0.087
25	P3	-0.974	0.594	-1.438	0.404	-2.376	0.190	-2.293	0.085
26	A1	-1.605	0.380	-1.673	0.404	-1.628	0.190	-3.053	0.078
27	T5	-1.739	0.354	-1.046	0.426	-2.119	0.190	-2.426	0.085
28	P1	-0.850	0.623	-1.267	0.404	-0.972	0.340	-0.612	0.552

29	P9	-2.300	0.316	-0.858	0.467	-2.241	0.190	-2.302	0.085
30	PO3	-0.839	0.623	-1.302	0.404	-1.104	0.303	-0.620	0.552
31	Pz	-0.649	0.709	-1.338	0.404	-1.016	0.324	-0.894	0.411
32	O1	-1.705	0.354	-0.349	0.782	-1.805	0.190	-1.044	0.349
33	POz	-0.895	0.623	-1.372	0.404	-0.891	0.376	-0.451	0.652
34	Oz	-2.192	0.316	-0.662	0.578	-1.214	0.266	-0.920	0.403
35	PO4	-0.399	0.803	-0.893	0.454	-1.023	0.324	-0.815	0.449
36	O2	-1.870	0.316	0.092	0.926	-1.636	0.190	-1.537	0.196
37	P2	-1.763	0.354	-1.224	0.404	-1.609	0.190	-1.399	0.232
38	CP2	-0.621	0.711	-0.258	0.820	-1.507	0.190	-0.669	0.534
39	P4	-1.128	0.536	-1.182	0.404	-1.924	0.190	-1.897	0.119
40	P10	-1.983	0.316	-0.298	0.808	-2.169	0.190	-2.031	0.098
41	T6	-1.027	0.586	0.247	0.820	-2.353	0.190	-2.464	0.085
42	P6	-1.515	0.384	-0.904	0.454	-1.700	0.190	-1.714	0.153
43	CP6	-1.304	0.468	-1.359	0.404	-1.669	0.190	-1.343	0.238
44	A2	-2.772	0.316	-1.397	0.404	-1.949	0.190	-2.850	0.078
45	TP8	-2.418	0.316	-1.247	0.404	-1.554	0.190	-2.127	0.092
46	C6	-1.548	0.380	-1.075	0.418	-1.639	0.190	-1.822	0.128
47	C4	-1.223	0.495	-0.894	0.454	-1.688	0.190	-2.078	0.092
48	C2	-0.491	0.741	-0.761	0.522	-1.697	0.190	-1.422	0.228
49	T4	-2.106	0.316	-1.092	0.418	-1.424	0.211	-2.627	0.078
50	FC4	-0.981	0.594	-1.006	0.431	-1.618	0.190	-1.346	0.238
51	FC2	-0.495	0.741	-0.632	0.580	-1.283	0.242	-1.300	0.250
52	FT8	-2.386	0.316	-2.243	0.404	-1.699	0.190	-1.360	0.238
53	FC6	-1.554	0.380	-1.758	0.404	-1.382	0.218	-1.206	0.276
54	F8	-1.306	0.468	-2.330	0.404	-1.526	0.190	-2.801	0.078
55	F6	-0.670	0.709	-1.893	0.404	-1.818	0.190	-1.607	0.184
56	F4	0.524	0.741	-1.616	0.404	-1.426	0.211	-2.527	0.085
57	Cz	-0.651	0.709	-0.901	0.454	-1.125	0.302	-1.230	0.270

Supplementary Table S2. Paired t-test results for comparison between rest period and working memory performance (theta frequency band).

		Group 1. Depressive disorder				Group 2. Anxiety disorder			
Electrodes		Absolute		Relative		Absolute		Relative	
No.	Name	t	p (FDR)	t	p (FDR)	t	p (FDR)	t	p (FDR)
01	AF4	0.674	0.734	-0.879	0.909	-1.841	0.149	-0.998	0.707
02	F2	0.078	0.956	-1.087	0.909	-2.870	0.086	-1.161	0.665
03	FCz	-2.199	0.182	-0.478	0.909	-2.317	0.125	-1.241	0.665
04	Fp2	0.345	0.868	-0.618	0.909	-1.374	0.239	-1.371	0.665
05	Fz	-0.207	0.908	-2.106	0.909	-1.476	0.212	-2.010	0.665
06	FC1	-0.359	0.868	0.991	0.909	-1.601	0.200	-0.960	0.720
07	AFz	0.225	0.908	-0.603	0.909	-2.758	0.086	-1.611	0.665
08	F1	-0.926	0.684	-1.001	0.909	-3.142	0.086	-1.515	0.665
09	Fp1	0.197	0.908	-0.411	0.909	-1.908	0.149	-1.947	0.665
10	AF3	0.260	0.906	0.124	0.969	-1.949	0.149	-0.881	0.735
11	F3	0.855	0.701	0.827	0.909	-1.162	0.291	-1.586	0.665
12	F5	-0.771	0.701	0.937	0.909	-2.197	0.141	-1.038	0.692
13	FC5	-0.394	0.860	0.908	0.909	-2.098	0.143	-0.769	0.744
14	FC3	-0.416	0.859	1.120	0.909	-1.369	0.239	-0.307	0.804
15	C1	0.010	0.991	1.058	0.909	-1.269	0.254	-0.276	0.804
16	F7	-0.834	0.701	0.863	0.909	-1.313	0.252	-0.316	0.804
17	FT7	-1.472	0.430	0.939	0.909	-1.595	0.200	-0.872	0.735
18	C3	-0.310	0.881	1.610	0.909	-1.538	0.205	0.397	0.804
19	CP1	0.075	0.956	0.504	0.909	-1.190	0.284	-0.749	0.744
20	C5	-0.720	0.730	1.412	0.909	-1.872	0.149	-0.357	0.804
21	T3	-1.559	0.415	0.850	0.909	-1.056	0.312	1.157	0.665
22	TP7	-1.375	0.468	0.645	0.909	-1.773	0.166	0.278	0.804
23	CP5	-0.779	0.701	1.023	0.909	-1.968	0.149	-0.520	0.804
24	P5	-1.339	0.468	0.478	0.909	-2.111	0.143	-0.506	0.804
25	P3	-0.688	0.734	0.027	0.986	-2.417	0.109	-0.647	0.801
26	A1	-1.589	0.415	-0.445	0.909	-1.112	0.299	-0.551	0.804
27	T5	-2.194	0.182	-0.699	0.909	-1.721	0.172	-0.528	0.804
28	P1	-1.049	0.653	-0.669	0.909	-1.353	0.240	-1.132	0.665

29	P9	-2.655	0.182	-0.470	0.909	-1.921	0.149	-0.362	0.804
30	PO3	-0.959	0.684	-0.945	0.909	-1.435	0.223	-1.915	0.665
31	Pz	-0.569	0.794	-0.848	0.909	-1.080	0.306	-1.424	0.665
32	O1	-2.537	0.182	0.215	0.909	-3.016	0.086	-1.317	0.665
33	POz	-0.922	0.684	-0.510	0.909	-1.147	0.292	-1.731	0.665
34	Oz	-2.770	0.182	0.048	0.986	-2.107	0.143	-1.590	0.665
35	PO4	-0.422	0.859	-0.309	0.909	-1.275	0.254	-1.938	0.665
36	O2	-2.255	0.182	0.217	0.909	-2.702	0.086	-1.080	0.676
37	P2	-1.645	0.413	-0.036	0.986	-2.413	0.109	-1.487	0.665
38	CP2	-1.246	0.516	-0.281	0.909	-1.855	0.149	-1.292	0.665
39	P4	-1.489	0.430	-0.696	0.909	-2.712	0.086	-1.291	0.665
40	P10	-2.357	0.182	-0.313	0.909	-2.237	0.138	-0.322	0.804
41	T6	-2.066	0.223	-0.593	0.909	-2.412	0.109	-0.692	0.778
42	P6	-1.785	0.347	-0.655	0.909	-2.428	0.109	-1.181	0.665
43	CP6	-1.453	0.430	-0.705	0.909	-2.020	0.149	-1.528	0.665
44	A2	-2.399	0.182	0.656	0.909	-1.283	0.254	-0.277	0.804
45	TP8	-2.631	0.182	-0.341	0.909	-1.542	0.205	-0.897	0.735
46	C6	-1.950	0.265	-0.225	0.909	-1.676	0.182	-1.118	0.665
47	C4	-1.628	0.413	-0.839	0.909	-1.561	0.205	-0.350	0.804
48	C2	-0.494	0.825	0.430	0.909	-1.734	0.172	-1.272	0.665
49	T4	-1.113	0.615	0.765	0.909	-1.001	0.321	0.386	0.804
50	FC4	-0.812	0.701	-0.473	0.909	-1.969	0.149	-0.785	0.744
51	FC2	-0.886	0.697	-0.472	0.909	-1.882	0.149	-0.841	0.743
52	FT8	-2.290	0.182	-0.017	0.986	-1.486	0.212	-0.538	0.804
53	FC6	-1.328	0.468	-0.220	0.909	-1.487	0.212	-0.497	0.804
54	F8	-0.616	0.769	0.504	0.909	-1.027	0.317	0.496	0.804
55	F6	-0.504	0.825	-0.357	0.909	-1.994	0.149	-0.754	0.744
56	F4	0.981	0.684	-0.277	0.909	-1.021	0.317	-0.107	0.916
57	Cz	-0.158	0.923	1.212	0.909	-1.108	0.299	-0.268	0.804

Supplementary Table S3. Paired t-test results for comparison between rest period and working memory performance (alpha frequency band).

		Group 1. Depressive disorder				Group 2. Anxiety disorder			
Electrodes		Absolute		Relative		Absolute		Relative	
No.	Name	t	p (FDR)	t	p (FDR)	t	p (FDR)	t	p (FDR)
01	AF4	1.030	0.688	1.283	0.510	-1.226	0.543	0.968	0.400
02	F2	0.229	0.881	0.954	0.513	-1.948	0.543	0.994	0.394
03	FCz	-1.907	0.388	0.924	0.513	-1.363	0.543	1.455	0.323
04	Fp2	1.395	0.564	1.895	0.510	-1.127	0.543	1.173	0.355
05	Fz	0.858	0.688	1.224	0.510	-1.260	0.543	1.255	0.343
06	FC1	0.098	0.922	1.275	0.510	-0.484	0.665	2.136	0.285
07	AFz	1.011	0.688	1.515	0.510	-1.045	0.543	1.408	0.323
08	F1	-0.361	0.837	1.296	0.510	-1.249	0.543	1.702	0.285
09	Fp1	1.653	0.429	1.003	0.510	-1.547	0.543	2.030	0.285
10	AF3	0.855	0.688	1.370	0.510	-1.337	0.543	1.997	0.285
11	F3	1.141	0.688	1.507	0.510	-1.054	0.543	0.899	0.416
12	F5	-0.851	0.688	2.199	0.510	-1.028	0.543	1.249	0.343
13	FC5	-0.371	0.837	1.109	0.510	-0.650	0.603	1.498	0.323
14	FC3	-0.167	0.900	1.016	0.510	-0.135	0.893	1.790	0.285
15	C1	0.381	0.837	1.105	0.510	-0.564	0.630	1.753	0.285
16	F7	-0.112	0.922	1.295	0.510	-1.431	0.543	1.021	0.394
17	FT7	-1.109	0.688	1.305	0.510	-0.696	0.594	1.123	0.370
18	C3	-0.454	0.837	0.921	0.513	-0.617	0.603	1.864	0.285
19	CP1	0.332	0.845	1.387	0.510	-0.739	0.590	1.291	0.340
20	C5	-0.531	0.831	1.002	0.510	-1.089	0.543	1.647	0.285
21	T3	-1.257	0.676	1.031	0.510	-1.232	0.543	1.612	0.291
22	TP7	-0.877	0.688	1.111	0.510	-1.202	0.543	1.686	0.285
23	CP5	-0.459	0.837	1.230	0.510	-0.889	0.543	2.069	0.285
24	P5	-0.949	0.688	0.999	0.510	-1.218	0.543	2.148	0.285
25	P3	-0.376	0.837	1.130	0.510	-1.647	0.543	1.801	0.285
26	A1	-0.798	0.704	1.517	0.510	0.235	0.830	2.474	0.285
27	T5	-1.467	0.535	0.979	0.511	-1.297	0.543	2.206	0.285
28	P1	-0.407	0.837	1.582	0.510	-0.996	0.543	0.949	0.403

29	P9	-2.138	0.341	0.523	0.701	-0.625	0.603	1.963	0.285
30	PO3	-0.293	0.861	1.461	0.510	-0.994	0.543	1.019	0.394
31	Pz	-0.247	0.881	1.351	0.510	-0.944	0.543	0.651	0.546
32	O1	-2.110	0.341	0.648	0.630	-0.775	0.585	1.317	0.340
33	POz	-0.464	0.837	1.410	0.510	-0.735	0.590	0.522	0.614
34	Oz	-1.832	0.398	1.081	0.510	-0.873	0.543	1.000	0.394
35	PO4	0.166	0.900	1.262	0.510	-0.500	0.665	0.912	0.416
36	O2	-2.080	0.341	0.143	0.903	-1.449	0.543	1.213	0.345
37	P2	-0.936	0.688	1.106	0.510	-0.865	0.543	1.445	0.323
38	CP2	-1.800	0.398	-0.353	0.795	-0.992	0.543	0.561	0.598
39	P4	-0.914	0.688	1.389	0.510	-1.315	0.543	1.701	0.285
40	P10	-2.141	0.341	-0.330	0.799	-1.228	0.543	1.214	0.345
41	T6	-2.371	0.341	-0.300	0.808	-0.967	0.543	1.475	0.323
42	P6	-1.007	0.688	0.870	0.526	-1.135	0.543	1.337	0.340
43	CP6	-0.697	0.732	1.414	0.510	-1.133	0.543	1.469	0.323
44	A2	-1.676	0.429	1.077	0.510	0.251	0.830	1.800	0.285
45	TP8	-2.018	0.343	0.905	0.513	-0.709	0.594	1.769	0.285
46	C6	-2.080	0.341	0.841	0.535	-1.154	0.543	1.368	0.335
47	C4	-1.644	0.429	0.672	0.630	-1.169	0.543	1.287	0.340
48	C2	-1.144	0.688	-0.021	0.983	-0.671	0.600	1.661	0.285
49	T4	-1.458	0.535	0.167	0.899	-0.913	0.543	2.188	0.285
50	FC4	-0.776	0.704	0.650	0.630	-1.269	0.543	0.806	0.464
51	FC2	-0.748	0.704	0.406	0.767	-0.789	0.585	1.410	0.323
52	FT8	-2.167	0.341	1.391	0.510	-1.224	0.543	1.164	0.355
53	FC6	-1.018	0.688	1.142	0.510	-1.594	0.543	0.284	0.778
54	F8	-0.640	0.766	0.563	0.684	-1.038	0.543	1.957	0.285
55	F6	-0.535	0.831	1.137	0.510	-1.717	0.543	0.765	0.481
56	F4	1.082	0.688	0.728	0.608	-0.935	0.543	1.098	0.370
57	Cz	-0.763	0.704	0.447	0.749	-1.091	0.543	1.093	0.370

Supplementary Table S4. Paired t-test results for comparison between rest period and working memory performance (beta frequency band).

		Group 1. Depressive disorder				Group 2. Anxiety disorder			
Electrodes		Absolute		Relative		Absolute		Relative	
No.	Name	t	p (FDR)	t	p (FDR)	t	p (FDR)	t	p (FDR)
01	AF4	1.558	0.820	2.627	0.087	-0.415	0.717	2.278	**0.049***
02	F2	1.225	0.820	2.161	0.175	-1.289	0.533	2.463	**0.037***
03	FCz	-0.702	0.910	2.054	0.180	-2.21	0.354	0.537	0.593
04	Fp2	1.27	0.820	1.982	0.189	-1.05	0.533	2.533	**0.035***
05	Fz	1.364	0.820	2.291	0.145	-1.144	0.533	1.795	0.114
06	FC1	0.621	0.910	0.393	0.696	-1.149	0.533	1.592	0.148
07	AFz	1.263	0.820	1.955	0.189	-0.853	0.539	2.066	0.070
08	F1	1.175	0.820	1.784	0.211	-1.274	0.533	2.955	**0.022***
09	Fp1	1.562	0.820	1.106	0.339	-1.747	0.451	2.723	**0.023***
10	AF3	1.972	0.820	1.528	0.250	-1.096	0.533	2.512	**0.035***
11	F3	1.875	0.820	2.1	0.175	-0.798	0.542	3.254	**0.018***
12	F5	0.053	0.975	1.947	0.189	-0.858	0.539	2.93	**0.022***
13	FC5	0.833	0.894	1.385	0.269	-0.728	0.546	3.162	**0.018***
14	FC3	0.639	0.910	0.855	0.442	-0.416	0.717	2.86	**0.022***
15	C1	1.064	0.820	1.551	0.250	-0.792	0.542	2.785	**0.023***
16	F7	1.039	0.820	2.85	0.068	-0.969	0.533	4.298	**0.002***
17	FT7	0.275	0.966	1.741	0.211	-0.543	0.646	2.408	**0.041***
18	C3	0.469	0.961	0.834	0.443	-0.786	0.542	2.828	**0.023***
19	CP1	1.139	0.820	1.199	0.312	-0.326	0.759	3.106	**0.019***
20	C5	0.216	0.966	1.019	0.371	-1.294	0.533	2.082	0.070
21	T3	-0.988	0.820	1.352	0.269	-0.853	0.539	1.662	0.138
22	TP7	0.136	0.975	1.641	0.230	-1.57	0.463	1.629	0.141
23	CP5	0.274	0.966	1.219	0.309	-1.256	0.533	2.277	**0.049***
24	P5	0.185	0.966	1.113	0.339	-1.798	0.451	2.325	**0.046***
25	P3	0.735	0.910	1.045	0.364	-1.805	0.451	3.248	**0.018***
26	A1	0.19	0.966	1.826	0.211	-0.339	0.759	1.63	0.141
27	T5	-0.283	0.966	1.626	0.230	-1.769	0.451	1.988	0.082
28	P1	0.274	0.966	0.695	0.507	-1.289	0.533	1.531	0.158

29	P9	-0.956	0.820	1.41	0.269	-1.066	0.533	1.02	0.329
30	PO3	0.722	0.910	1.358	0.269	-1.205	0.533	1.535	0.158
31	Pz	1.144	0.820	2.13	0.175	-1.04	0.533	1.91	0.092
32	O1	-1.589	0.820	0.476	0.647	-2.377	0.338	1.059	0.316
33	POz	0.717	0.910	1.772	0.211	-1.107	0.533	1.358	0.205
34	Oz	-1.153	0.820	0.769	0.470	-2.51	0.338	0.85	0.413
35	PO4	0.846	0.894	0.827	0.443	-1.11	0.533	1.34	0.207
36	O2	-1.581	0.820	0.902	0.423	-2.378	0.338	1.923	0.092
37	P2	0.022	0.982	1.538	0.250	-1.997	0.451	1.515	0.158
38	CP2	-0.172	0.966	1.233	0.309	-0.945	0.537	1.284	0.224
39	P4	0.185	0.966	1.329	0.269	-2.323	0.338	2.106	0.068
40	P10	-0.315	0.966	2.691	0.087	-1.575	0.463	3.205	**0.018***
41	T6	-0.616	0.910	1.161	0.324	-1.697	0.451	4.263	**0.002***
42	P6	0.053	0.975	1.388	0.269	-1.736	0.451	2.739	**0.023***
43	CP6	0.292	0.966	1.709	0.211	-1.631	0.463	2.337	**0.046***
44	A2	-1.303	0.820	1.378	0.269	0.087	0.931	2.884	**0.022***
45	TP8	-0.612	0.910	1.74	0.211	-0.759	0.546	3.252	**0.018***
46	C6	-0.498	0.961	1.331	0.269	-0.98	0.533	3.362	**0.018***
47	C4	-0.116	0.975	1.476	0.267	-0.987	0.533	3.029	**0.021***
48	C2	0.335	0.966	1.434	0.269	-1.144	0.533	1.513	0.158
49	T4	0.09	0.975	2.539	0.087	-1.088	0.533	1.673	0.138
50	FC4	0.951	0.820	1.778	0.211	-0.887	0.539	2.789	**0.023***
51	FC2	0.471	0.961	1.712	0.211	-1.464	0.530	0.787	0.442
52	FT8	0.422	0.966	3.713	**0.013***	-0.782	0.542	2.19	0.058
53	FC6	1.021	0.820	2.579	0.087	-0.569	0.639	2.545	**0.035***
54	F8	1.724	0.820	4.663	**0.001***	-0.919	0.539	2.481	**0.037***
55	F6	1.236	0.820	3.193	**0.032***	-0.738	0.546	2.76	**0.023***
56	F4	2.141	0.820	3.581	**0.013***	-0.687	0.564	2.88	**0.022***
57	Cz	0.528	0.961	0.962	0.396	-1.003	0.533	1.738	0.125

Supplementary Table S5. Paired t-test results for comparison between rest period and working memory performance (gamma frequency band).

		Group 1. Depressive disorder				Group 2. Anxiety disorder			
Electrodes		Absolute		Relative		Absolute		Relative	
No.	Name	t	p (FDR)	t	p (FDR)	t	p (FDR)	t	p (FDR)
01	AF4	1.129	0.976	0.978	0.915	-0.499	0.654	1.29	0.827
02	F2	0.955	0.976	0.872	0.915	-0.69	0.565	1.925	0.827
03	FCz	-1.69	0.976	-0.497	0.931	-1.955	0.456	-0.382	0.965
04	Fp2	0.399	0.976	-1.09	0.915	-1.174	0.456	0.271	0.965
05	Fz	0.532	0.976	-0.37	0.967	-1.192	0.456	1.004	0.827
06	FC1	0.108	0.976	-0.919	0.915	-1.141	0.456	0.117	0.976
07	AFz	0.896	0.976	0.375	0.967	-1.245	0.456	0.69	0.827
08	F1	0.086	0.976	-0.163	0.976	-1.365	0.456	1.461	0.827
09	Fp1	-0.711	0.976	-1.758	0.915	-1.781	0.456	-0.023	0.982
10	AF3	0.01	0.992	-0.58	0.915	-1.288	0.456	1.012	0.827
11	F3	-0.35	0.976	-1.345	0.915	-0.986	0.456	1.312	0.827
12	F5	-1.267	0.976	-0.994	0.915	-0.727	0.565	1.049	0.827
13	FC5	-0.299	0.976	-0.82	0.915	-0.667	0.567	1.207	0.827
14	FC3	0.083	0.976	-0.614	0.915	-0.196	0.86	1.681	0.827
15	C1	0.344	0.976	-0.228	0.976	-0.531	0.643	0.999	0.827
16	F7	-0.926	0.976	-0.081	0.977	-1.228	0.456	0.224	0.965
17	FT7	-0.374	0.976	-0.207	0.976	-0.686	0.565	0.828	0.827
18	C3	0.051	0.976	-0.888	0.915	-0.760	0.563	1.295	0.827
19	CP1	0.726	0.976	0.379	0.967	-0.560	0.633	0.702	0.827
20	C5	0.174	0.976	-0.208	0.976	-1.242	0.456	0.518	0.96
21	T3	-1.303	0.976	-1.027	0.915	-1.039	0.456	-0.629	0.866
22	TP7	0.198	0.976	0.325	0.967	-1.538	0.456	0.267	0.965
23	CP5	0.253	0.976	0.048	0.977	-1.849	0.456	0.048	0.979
24	P5	0.169	0.976	0.675	0.915	-2.122	0.456	-0.172	0.965
25	P3	0.678	0.976	0.855	0.915	-2.446	0.456	0.070	0.979
26	A1	0.420	0.976	0.536	0.915	-0.280	0.809	1.219	0.827
27	T5	-0.570	0.976	-0.138	0.976	-1.703	0.456	0.159	0.965
28	P1	0.092	0.976	0.15	0.976	-1.375	0.456	-1.201	0.827

29	P9	-0.918	0.976	0.351	0.967	-0.702	0.565	1.277	0.827
30	PO3	0.524	0.976	-0.031	0.977	-1.114	0.456	0.199	0.965
31	Pz	0.568	0.976	-0.616	0.915	-0.998	0.456	0.338	0.965
32	O1	-2.156	0.668	-1.532	0.915	-2.079	0.456	-0.29	0.965
33	POz	0.327	0.976	-0.967	0.915	-0.753	0.563	0.999	0.827
34	Oz	-2.467	0.470	-2.108	0.915	-1.747	0.456	0.718	0.827
35	PO4	0.111	0.976	-1.371	0.915	-1.031	0.456	0.949	0.827
36	O2	-2.655	0.470	-2.34	0.915	-1.48	0.456	0.845	0.827
37	P2	-0.518	0.976	-0.622	0.915	-1.732	0.456	0.806	0.827
38	CP2	-0.097	0.976	0.547	0.915	-0.898	0.494	0.437	0.965
39	P4	-0.063	0.976	0.055	0.977	-1.728	0.456	0.904	0.827
40	P10	-1.424	0.976	-0.563	0.915	-1.69	0.456	0.253	0.965
41	T6	-1.523	0.976	-1.054	0.915	-1.484	0.456	0.734	0.827
42	P6	-0.531	0.976	0.177	0.976	-1.4	0.456	0.703	0.827
43	CP6	-0.189	0.976	0.59	0.915	-1.456	0.456	0.241	0.965
44	A2	-1.797	0.976	-0.342	0.967	-0.174	0.863	0.898	0.827
45	TP8	-1.159	0.976	0.816	0.915	-1.076	0.456	0.258	0.965
46	C6	-1.000	0.976	0.758	0.915	-1.033	0.456	0.740	0.827
47	C4	-0.509	0.976	0.893	0.915	-0.998	0.456	0.756	0.827
48	C2	0.249	0.976	0.545	0.915	-1.411	0.456	0.079	0.979
49	T4	-1.609	0.976	-0.613	0.915	-1.316	0.456	-0.846	0.827
50	FC4	0.288	0.976	0.619	0.915	-1.203	0.456	0.396	0.965
51	FC2	-0.148	0.976	0.03	0.977	-1.236	0.456	0.151	0.965
52	FT8	-0.537	0.976	1.115	0.915	-1.516	0.456	-0.325	0.965
53	FC6	0.413	0.976	1.187	0.915	-0.867	0.505	0.882	0.827
54	F8	0.373	0.976	1.661	0.915	-1.27	0.456	-0.991	0.827
55	F6	0.654	0.976	1.227	0.915	-0.921	0.489	1.197	0.827
56	F4	1.082	0.976	0.269	0.976	-1.001	0.456	1.198	0.827
57	Cz	-0.172	0.976	-1.392	0.915	-1.136	0.456	-1.579	0.827

Supplementary Table S6. Correlation analysis result of delta absolute power and LF-HRV.

		Group 1. Depressive disorder				Group 2. Anxiety disorder			
Electrodes		Resting		Task		Resting		Task	
No.	Name	r	p (FDR)	r	p (FDR)	r	p (FDR)	r	p (FDR)
01	AF4	-0.194	0.643	0.408	**0.006***	0.036	0.862	-0.165	0.457
02	F2	-0.173	0.665	0.445	**0.002***	-0.047	0.875	-0.163	0.708
03	FCz	0.048	0.726	0.305	0.072	0.014	0.956	-0.030	0.957
04	Fp2	-0.176	0.482	0.341	**0.033***	0.059	0.846	-0.104	0.653
05	Fz	-0.148	0.512	0.407	**0.008***	-0.014	0.951	-0.102	0.684
06	FC1	-0.187	0.612	0.238	0.253	0.131	0.950	0.030	0.947
07	AFz	-0.239	0.248	0.502	**0.001***	0.067	0.794	-0.023	0.982
08	F1	-0.075	0.948	0.355	**0.022***	0.114	0.895	-0.088	0.854
09	Fp1	-0.020	0.958	0.356	**0.023***	0.072	0.956	-0.108	0.575
10	AF3	-0.085	0.989	0.416	**0.006***	0.076	0.843	-0.114	0.761
11	F3	-0.005	0.995	0.339	**0.033***	0.020	0.923	-0.129	0.690
12	F5	-0.048	0.887	0.365	**0.018***	0.130	0.918	-0.142	0.664
13	FC5	-0.043	0.892	0.359	**0.017***	0.091	0.915	-0.148	0.743
14	FC3	-0.117	0.779	0.348	**0.028***	0.092	0.984	-0.026	1.000
15	C1	-0.138	0.700	0.327	0.060	0.112	0.861	0.003	0.983
16	F7	0.125	0.516	0.295	0.089	0.074	0.971	-0.147	0.707
17	FT7	-0.008	0.968	0.377	**0.018***	0.036	0.938	-0.127	0.885
18	C3	-0.125	0.863	0.337	**0.048***	0.062	0.812	-0.097	0.976
19	CP1	-0.142	0.622	0.296	0.087	0.151	0.893	0.021	0.946
20	C5	-0.023	0.865	0.378	**0.014***	0.035	0.862	-0.004	0.986
21	T3	0.149	0.497	0.154	0.479	0.005	0.969	-0.104	0.913
22	TP7	-0.029	0.831	0.363	**0.019***	0.079	0.867	-0.025	0.871
23	CP5	-0.054	0.951	0.387	**0.014***	0.089	0.854	-0.006	0.969
24	P5	-0.037	0.992	0.412	**0.008***	0.189	0.783	0.036	0.927
25	P3	-0.053	0.994	0.391	**0.012***	0.147	0.908	0.020	0.957
26	A1	0.005	0.974	0.261	0.269	0.054	0.891	-0.018	0.946
27	T5	-0.083	0.929	0.251	0.201	0.154	0.900	-0.023	0.920
28	P1	-0.055	0.812	0.336	**0.049***	0.032	0.953	-0.019	0.963
29	P9	0.002	0.987	0.315	0.059	0.192	0.629	0.045	0.986

30	PO3	-0.109	0.612	0.362	**0.026***	0.124	0.782	-0.019	0.928
31	Pz	-0.095	0.789	0.298	0.111	0.123	0.829	-0.065	0.900
32	O1	-0.006	0.963	0.254	0.154	0.148	0.603	0.052	0.980
33	POz	-0.100	0.642	0.334	0.052	0.156	0.679	-0.047	0.940
34	Oz	-0.014	0.918	0.201	0.450	0.175	0.503	0.002	0.991
35	PO4	0.030	0.942	0.377	**0.018***	0.149	0.613	-0.040	0.955
36	O2	-0.016	0.962	0.245	0.222	0.186	0.388	-0.017	0.997
37	P2	0.015	0.912	0.393	**0.016***	0.065	0.702	-0.063	0.848
38	CP2	0.016	0.907	0.426	**0.005***	0.099	0.851	-0.030	0.980
39	P4	-0.041	0.893	0.397	**0.011***	0.103	0.802	-0.093	0.979
40	P10	-0.092	0.913	0.298	0.083	0.126	0.897	-0.031	0.932
41	T6	-0.012	0.940	0.373	**0.015***	0.158	0.785	-0.031	0.894
42	P6	-0.021	0.990	0.409	**0.007***	0.152	0.976	-0.007	0.997
43	CP6	-0.004	0.975	0.406	**0.008***	0.203	0.637	-0.011	0.954
44	A2	-0.017	0.938	0.356	**0.034***	0.174	0.966	0.006	0.967
45	TP8	0.062	0.914	0.383	**0.015***	0.107	0.968	-0.054	0.925
46	C6	0.033	0.993	0.412	**0.007***	0.122	0.949	-0.066	0.978
47	C4	0.000	0.999	0.316	0.058	0.129	0.823	-0.062	0.997
48	C2	-0.065	0.747	0.283	0.113	0.030	0.896	-0.041	0.910
49	T4	0.205	0.561	0.321	0.051	0.115	0.792	-0.127	0.850
50	FC4	0.030	0.893	0.397	**0.008***	0.021	0.882	-0.107	0.871
51	FC2	-0.024	0.947	0.230	0.274	-0.026	0.920	-0.063	0.966
52	FT8	0.023	0.892	0.312	0.063	0.026	0.977	-0.174	0.693
53	FC6	0.036	0.885	0.408	**0.006***	0.014	0.918	-0.148	0.837
54	F8	0.097	0.698	0.291	0.077	0.049	0.966	-0.153	0.773
55	F6	-0.063	0.944	0.298	0.083	-0.009	0.969	-0.201	0.413
56	F4	-0.070	0.852	0.423	**0.004***	0.002	0.989	-0.162	0.541
57	Cz	-0.007	0.962	0.318	0.056	0.079	0.917	-0.044	0.945

Supplementary Table S7. Correlation analysis result of delta relative power and LF-HRV.

Electrodes		Group 1. Depressive disorder				Group 2. Anxiety disorder			
		Resting		Task		Resting		Task	
No.	Name	r	p (FDR)	r	p (FDR)	r	p (FDR)	r	p (FDR)
01	AF4	-0.070	0.831	0.279	0.315	0.071	0.935	-0.020	0.962
02	F2	-0.062	0.848	0.270	0.272	0.065	0.873	0.003	0.981
03	FCz	0.012	0.955	0.266	0.204	-0.043	0.820	0.109	0.966
04	Fp2	-0.098	0.960	0.305	0.142	0.131	0.986	-0.029	0.960
05	Fz	-0.036	0.981	0.336	0.132	0.039	0.968	0.071	0.984
06	FC1	-0.012	0.982	0.265	0.209	-0.062	0.655	0.033	0.993
07	AFz	-0.142	0.940	0.397	**0.032***	0.059	0.872	0.095	0.966
08	F1	-0.047	0.865	0.311	0.206	0.094	0.648	0.063	0.880
09	Fp1	-0.048	0.978	0.393	**0.036***	0.104	0.980	0.018	0.984
10	AF3	0.025	0.931	0.374	0.058	0.126	0.858	0.033	0.891
11	F3	0.102	0.892	0.315	0.091	0.057	0.909	-0.041	0.961
12	F5	0.077	0.899	0.312	0.140	0.092	0.875	0.017	0.906
13	FC5	0.045	0.875	0.251	0.269	0.034	0.805	-0.015	0.948
14	FC3	0.031	0.977	0.339	0.069	-0.014	0.921	0.035	0.962
15	C1	0.078	0.858	0.388	**0.021***	-0.070	0.616	0.039	0.885
16	F7	0.161	0.551	0.344	0.091	-0.006	0.968	-0.003	0.991
17	FT7	0.064	0.868	0.285	0.222	0.026	0.978	0.009	0.956
18	C3	0.087	0.786	0.417	**0.009***	-0.016	0.907	0.005	0.969
19	CP1	0.073	0.815	0.399	**0.025***	-0.008	0.953	0.070	0.778
20	C5	0.085	0.732	0.394	**0.014***	-0.044	0.816	0.039	0.921
21	T3	0.089	0.885	0.289	0.100	-0.108	0.708	0.072	0.935
22	TP7	0.041	0.792	0.413	**0.010***	-0.001	0.994	0.037	0.925
23	CP5	0.035	0.904	0.422	**0.005***	-0.017	0.904	0.012	0.932
24	P5	0.024	0.985	0.422	**0.008***	0.052	0.770	0.044	0.831
25	P3	0.029	0.950	0.376	**0.026***	0.042	0.765	0.055	0.821
26	A1	0.049	0.957	0.365	0.073	-0.074	0.702	-0.005	0.974
27	T5	-0.026	0.920	0.342	0.056	0.060	0.724	0.035	0.901
28	P1	0.025	0.999	0.298	0.168	-0.012	0.965	0.120	0.777
29	P9	-0.040	0.911	0.376	**0.033***	0.032	0.885	0.042	0.901

30	PO3	0.098	0.902	0.356	0.091	-0.010	0.943	0.087	0.989
31	Pz	0.144	0.662	0.366	0.072	-0.036	0.939	0.049	0.954
32	O1	-0.035	0.994	0.229	0.193	-0.013	0.925	0.034	0.874
33	POz	0.117	0.845	0.356	0.089	-0.029	0.861	0.059	0.975
34	Oz	0.003	0.982	0.255	0.124	0.011	0.937	0.047	0.991
35	PO4	0.083	0.945	0.329	0.070	-0.002	0.990	0.077	0.935
36	O2	-0.039	0.973	0.220	0.269	-0.014	0.927	0.032	0.966
37	P2	0.019	0.920	0.283	0.140	0.054	0.874	0.053	0.943
38	CP2	-0.044	0.941	0.285	0.118	-0.025	0.858	0.084	0.931
39	P4	-0.014	0.917	0.285	0.109	0.018	0.900	-0.012	0.938
40	P10	-0.022	0.870	0.327	0.085	-0.079	0.929	-0.001	0.997
41	T6	-0.043	0.951	0.286	0.140	-0.024	0.976	0.002	0.990
42	P6	0.009	0.972	0.357	0.051	-0.022	0.874	-0.006	0.964
43	CP6	-0.021	0.930	0.328	0.082	0.019	0.893	-0.028	0.911
44	A2	0.054	0.883	0.354	0.076	-0.028	0.842	-0.009	0.999
45	TP8	0.050	0.958	0.334	0.076	-0.009	0.949	-0.070	0.800
46	C6	-0.010	0.966	0.336	**0.049***	0.018	0.898	-0.067	0.822
47	C4	-0.020	0.989	0.248	0.248	0.014	0.920	-0.048	0.774
48	C2	-0.032	0.973	0.327	0.091	-0.004	0.980	0.056	0.918
49	T4	0.134	0.706	0.407	**0.020***	0.028	0.839	-0.020	0.966
50	FC4	-0.064	0.829	0.240	0.309	-0.027	0.896	-0.015	0.914
51	FC2	-0.016	0.914	0.247	0.255	-0.028	0.843	0.061	0.918
52	FT8	0.052	0.872	0.287	0.155	0.062	0.983	-0.096	0.797
53	FC6	-0.001	0.993	0.266	0.259	0.064	0.837	-0.019	0.890
54	F8	0.179	0.515	0.328	0.062	0.027	0.964	-0.119	0.637
55	F6	0.010	0.991	0.250	0.261	0.063	0.963	-0.083	0.838
56	F4	0.053	0.889	0.320	0.170	0.063	0.908	-0.102	0.837
57	Cz	0.042	0.977	0.432	**0.011***	-0.040	0.840	0.095	0.777

Supplementary Table S8. Correlation analysis result of theta absolute power and LF-HRV.

		Group 1. Depressive disorder				Group 2. Anxiety disorder			
Electrodes		Resting		Task		Resting		Task	
No.	Name	r	p (FDR)	r	p (FDR)	r	p (FDR)	r	p (FDR)
01	AF4	-0.188	0.546	0.233	0.155	0.145	0.934	0.021	1.000
02	F2	-0.143	0.632	0.199	0.221	0.056	0.938	-0.001	0.996
03	FCz	0.037	0.884	0.134	0.382	0.133	0.922	0.063	0.916
04	Fp2	-0.139	0.570	0.199	0.231	0.212	0.866	-0.037	0.856
05	Fz	-0.108	0.698	0.137	0.369	0.141	0.831	-0.062	0.803
06	FC1	-0.153	0.686	0.063	0.927	0.206	0.706	0.133	0.823
07	AFz	-0.156	0.406	0.267	0.092	0.151	0.969	0.027	0.916
08	F1	-0.081	0.824	0.097	0.516	0.153	0.884	0.061	0.863
09	Fp1	-0.070	0.787	0.140	0.355	0.174	0.976	-0.045	0.839
10	AF3	-0.139	0.701	0.200	0.259	0.158	0.824	0.067	0.987
11	F3	-0.077	0.770	0.098	0.510	0.170	0.694	-0.042	0.965
12	F5	-0.130	0.738	0.127	0.458	0.193	0.742	0.088	0.951
13	FC5	-0.084	0.765	0.161	0.320	0.153	0.808	0.082	0.921
14	FC3	-0.146	0.689	0.111	0.477	0.158	0.913	0.147	0.879
15	C1	-0.142	0.676	0.080	0.688	0.132	0.848	0.089	0.840
16	F7	0.051	0.925	0.031	0.820	0.237	0.645	-0.055	0.910
17	FT7	-0.038	0.987	0.174	0.325	0.151	0.776	0.067	0.976
18	C3	-0.127	0.874	0.093	0.645	0.156	0.945	0.062	0.922
19	CP1	-0.118	0.748	0.052	0.814	0.177	0.802	0.090	0.873
20	C5	-0.020	0.918	0.166	0.332	0.164	0.902	0.113	0.877
21	T3	0.093	0.588	-0.050	0.847	0.254	0.599	-0.018	0.976
22	TP7	-0.024	0.927	0.119	0.553	0.188	0.855	0.101	0.943
23	CP5	-0.032	0.994	0.153	0.375	0.200	0.785	0.148	0.850
24	P5	-0.029	0.971	0.175	0.364	0.252	0.682	0.189	0.839
25	P3	-0.040	0.984	0.151	0.434	0.223	0.822	0.172	0.912
26	A1	0.016	0.906	0.095	0.678	0.228	0.645	0.138	0.865
27	T5	-0.056	0.890	0.045	0.805	0.220	0.726	0.138	0.919
28	P1	-0.029	0.914	0.121	0.541	0.181	0.913	0.085	0.784
29	P9	0.007	0.958	0.097	0.519	0.258	0.541	0.192	0.770

30	PO3	-0.138	0.404	0.097	0.563	0.223	0.567	0.057	0.902
31	Pz	-0.177	0.499	0.020	0.952	0.246	0.625	-0.030	0.981
32	O1	-0.037	0.788	0.070	0.692	0.263	0.358	0.226	0.924
33	POz	-0.175	0.507	0.052	0.760	0.245	0.495	0.061	0.957
34	Oz	-0.073	0.914	0.000	1.000	0.269	0.321	0.177	0.998
35	PO4	-0.072	0.928	0.086	0.747	0.245	0.481	0.042	0.927
36	O2	-0.044	0.951	0.046	0.800	0.288	0.226	0.202	0.772
37	P2	-0.046	0.988	0.122	0.542	0.175	0.661	0.110	0.883
38	CP2	0.010	0.943	0.165	0.418	0.164	0.764	0.063	0.977
39	P4	-0.070	0.781	0.132	0.430	0.213	0.787	0.133	0.854
40	P10	-0.122	0.835	0.016	0.904	0.290	0.433	0.195	0.774
41	T6	-0.044	0.746	0.093	0.535	0.260	0.546	0.203	0.697
42	P6	-0.059	0.887	0.091	0.548	0.262	0.592	0.211	0.739
43	CP6	-0.013	0.945	0.144	0.419	0.237	0.681	0.161	0.996
44	A2	-0.073	0.905	0.038	0.807	0.255	0.566	0.193	0.594
45	TP8	0.018	0.969	0.104	0.582	0.169	0.844	0.128	0.900
46	C6	0.035	0.990	0.151	0.385	0.162	0.825	0.095	0.998
47	C4	0.001	0.991	0.142	0.508	0.175	0.736	0.094	0.934
48	C2	-0.073	0.952	0.056	0.960	0.114	0.874	0.068	0.928
49	T4	0.088	0.655	0.043	0.817	0.259	0.431	-0.032	0.999
50	FC4	0.043	0.891	0.188	0.358	0.128	0.771	0.073	0.948
51	FC2	-0.039	0.994	0.046	0.962	0.102	0.891	0.044	0.834
52	FT8	0.030	0.961	0.102	0.654	0.106	0.912	0.051	0.986
53	FC6	0.050	0.881	0.203	0.249	0.128	0.845	0.055	0.972
54	F8	0.082	0.710	0.072	0.648	0.191	0.831	-0.046	0.922
55	F6	-0.044	0.997	0.132	0.450	0.130	0.911	0.071	0.927
56	F4	-0.074	0.861	0.175	0.258	0.132	0.990	-0.057	0.886
57	Cz	0.003	0.980	0.071	0.655	0.200	0.720	-0.011	0.939

Supplementary Table S9. Correlation analysis result of theta relative power and LF-HRV.

		Group 1. Depressive disorder				Group 2. Anxiety disorder			
Electrodes		Resting		Task		Resting		Task	
No.	Name	r	p (FDR)	r	p (FDR)	r	p (FDR)	r	p (FDR)
01	AF4	0.050	0.975	-0.054	0.923	0.224	0.367	0.376	**0.049***
02	F2	0.015	0.941	-0.059	0.868	0.208	0.453	0.339	0.069
03	FCz	0.046	0.977	0.003	0.989	0.198	0.652	0.200	0.529
04	Fp2	0.000	1.000	-0.070	0.811	0.286	0.389	0.389	**0.024***
05	Fz	0.001	0.992	-0.147	0.406	0.292	0.362	0.326	0.073
06	FC1	0.038	0.993	-0.008	0.954	0.146	0.726	0.193	0.432
07	AFz	0.125	0.956	-0.127	0.648	0.228	0.610	0.285	0.201
08	F1	0.004	0.998	-0.087	0.970	0.169	0.680	0.310	0.170
09	Fp1	-0.136	0.875	-0.088	0.843	0.229	0.627	0.363	0.050
10	AF3	-0.069	0.983	-0.076	0.923	0.188	0.626	0.361	0.061
11	F3	-0.122	0.773	-0.233	0.365	0.25	0.220	0.382	**0.045***
12	F5	-0.087	0.964	-0.152	0.854	0.157	0.828	0.340	0.085
13	FC5	-0.014	0.943	-0.104	0.869	0.145	0.771	0.303	0.160
14	FC3	0.051	0.987	-0.073	0.951	0.155	0.854	0.267	0.352
15	C1	0.141	0.804	-0.016	0.980	0.077	0.877	0.192	0.462
16	F7	-0.005	0.970	-0.262	0.219	0.243	0.342	0.379	0.062
17	FT7	0.065	0.955	-0.104	0.842	0.144	0.826	0.300	0.164
18	C3	0.149	0.790	0.023	0.999	0.151	0.815	0.254	0.278
19	CP1	0.175	0.740	0.041	0.972	0.091	0.869	0.149	0.666
20	C5	0.124	0.696	0.008	0.984	0.155	0.808	0.222	0.461
21	T3	0.092	0.780	-0.144	0.694	0.306	0.154	0.445	**0.010***
22	TP7	0.110	0.891	-0.029	0.913	0.169	0.729	0.254	0.275
23	CP5	0.140	0.745	0.029	0.991	0.176	0.808	0.247	0.328
24	P5	0.066	0.946	0.012	0.996	0.198	0.627	0.267	0.510
25	P3	0.091	0.955	0.024	0.998	0.182	0.854	0.277	0.399
26	A1	0.089	0.703	-0.016	0.910	0.213	0.751	0.298	0.370
27	T5	0.056	0.986	-0.009	0.948	0.196	0.732	0.290	0.437
28	P1	0.058	0.989	0.036	0.974	0.216	0.718	0.290	0.437
29	P9	0.057	0.934	0.013	0.967	0.193	0.804	0.280	0.522

30	PO3	0.013	0.973	-0.018	0.991	0.211	0.764	0.248	0.630
31	Pz	-0.038	0.986	-0.040	0.896	0.205	0.656	0.265	0.422
32	O1	-0.031	0.893	0.026	0.997	0.212	0.811	0.290	0.437
33	POz	-0.011	0.993	-0.022	0.952	0.213	0.657	0.267	0.614
34	Oz	-0.025	0.858	-0.015	0.914	0.22	0.725	0.302	0.346
35	PO4	-0.072	0.797	-0.033	0.917	0.187	0.832	0.227	0.885
36	O2	-0.068	0.741	-0.110	0.928	0.223	0.753	0.330	0.192
37	P2	-0.098	0.874	-0.086	0.838	0.179	0.866	0.295	0.394
38	CP2	0.003	0.983	-0.078	0.987	0.114	0.768	0.210	0.502
39	P4	-0.010	0.952	-0.095	0.910	0.211	0.564	0.352	0.074
40	P10	-0.031	0.978	-0.076	0.931	0.258	0.603	0.333	0.182
41	T6	-0.069	0.950	-0.175	0.854	0.211	0.649	0.329	0.195
42	P6	-0.008	0.955	-0.138	0.895	0.212	0.469	0.355	0.073
43	CP6	0.059	0.777	-0.106	0.947	0.196	0.557	0.356	0.053
44	A2	0.008	0.989	-0.069	0.962	0.185	0.722	0.305	0.293
45	TP8	0.060	0.923	-0.126	0.984	0.153	0.716	0.335	0.057
46	C6	0.080	0.933	-0.120	0.993	0.145	0.688	0.311	0.097
47	C4	0.076	0.927	-0.057	0.998	0.148	0.739	0.267	0.262
48	C2	0.079	0.930	-0.117	0.988	0.144	0.647	0.268	0.264
49	T4	0.090	0.872	-0.117	0.693	0.297	0.379	0.366	0.085
50	FC4	0.017	0.998	-0.088	0.994	0.18	0.501	0.335	0.126
51	FC2	0.033	0.996	-0.064	0.919	0.196	0.413	0.248	0.263
52	FT8	0.054	0.964	-0.152	0.856	0.16	0.538	0.414	**0.012***
53	FC6	0.013	0.941	-0.087	0.896	0.195	0.412	0.393	**0.024***
54	F8	0.156	0.759	-0.164	0.494	0.269	0.351	0.421	**0.020***
55	F6	0.042	0.990	-0.117	0.778	0.204	0.417	0.425	**0.018***
56	F4	0.017	0.986	-0.189	0.526	0.28	0.217	0.432	**0.007***
57	Cz	0.109	0.975	-0.025	0.941	0.204	0.515	0.283	0.384

Supplementary Table S10. Correlation analysis result of alpha absolute power and LF-HRV.

		Group 1. Depressive disorder				Group 2. Anxiety disorder			
Electrodes		Resting		Task		Resting		Task	
No.	Name	r	p (FDR)	r	p (FDR)	r	p (FDR)	r	p (FDR)
01	AF4	-0.104	0.727	0.096	0.557	-0.064	0.933	-0.281	0.304
02	F2	-0.043	0.813	0.097	0.528	-0.115	0.800	-0.271	0.320
03	FCz	0.030	0.904	0.083	0.543	-0.003	0.981	-0.118	0.907
04	Fp2	-0.038	0.779	0.084	0.584	-0.071	0.831	-0.209	0.534
05	Fz	-0.028	0.936	0.071	0.656	-0.108	0.969	-0.215	0.405
06	FC1	-0.118	0.717	0.010	0.940	0.115	0.761	-0.023	0.955
07	AFz	-0.052	0.775	0.091	0.549	-0.081	0.934	-0.271	0.307
08	F1	-0.003	0.986	0.020	0.883	-0.038	0.941	-0.255	0.600
09	Fp1	0.031	0.892	0.001	0.995	-0.062	0.909	-0.223	0.360
10	AF3	-0.040	0.912	0.025	0.855	-0.038	0.954	-0.291	0.213
11	F3	-0.006	0.967	0.067	0.624	0.036	0.951	-0.167	0.589
12	F5	-0.041	0.843	0.048	0.727	0.043	0.973	-0.213	0.791
13	FC5	-0.006	0.966	0.110	0.456	0.075	0.863	-0.143	0.969
14	FC3	-0.069	0.804	0.071	0.652	0.091	0.831	-0.063	0.942
15	C1	-0.164	0.428	-0.021	0.875	0.126	0.523	-0.014	0.985
16	F7	-0.004	0.976	0.011	0.935	0.120	0.888	-0.160	0.800
17	FT7	-0.015	0.913	0.095	0.528	0.071	0.878	-0.150	0.921
18	C3	-0.145	0.761	-0.034	0.801	0.095	0.786	-0.070	0.922
19	CP1	-0.186	0.646	-0.094	0.795	0.152	0.559	0.017	0.959
20	C5	0.006	0.968	0.107	0.513	0.120	0.839	-0.025	0.868
21	T3	0.008	0.986	-0.071	0.870	0.158	0.431	-0.114	0.975
22	TP7	0.009	0.947	0.077	0.619	0.113	0.721	-0.046	0.929
23	CP5	-0.006	0.968	0.093	0.568	0.155	0.694	0.008	0.985
24	P5	0.001	0.994	0.083	0.611	0.152	0.629	0.004	0.989
25	P3	-0.008	0.952	0.085	0.629	0.108	0.883	-0.054	0.955
26	A1	-0.015	0.921	0.020	0.954	0.149	0.457	-0.016	0.950
27	T5	-0.003	0.983	0.027	0.841	0.092	0.661	-0.044	0.964
28	P1	-0.027	0.843	0.003	0.981	-0.015	0.921	-0.127	0.878
29	P9	0.028	0.839	0.048	0.783	0.131	0.477	0.002	0.989

30	PO3	-0.060	0.779	0.003	0.983	0.047	0.991	-0.077	0.890
31	Pz	-0.102	0.845	-0.050	0.844	0.105	0.977	-0.063	0.953
32	O1	0.027	0.972	0.037	0.853	0.068	0.851	-0.028	0.939
33	POz	-0.112	0.665	-0.040	0.835	0.066	0.969	-0.120	0.894
34	Oz	-0.020	0.885	-0.009	0.970	0.068	0.930	-0.108	0.814
35	PO4	-0.001	0.996	0.008	0.952	0.065	0.943	-0.077	0.957
36	O2	0.030	0.892	0.038	0.854	0.076	0.977	-0.124	0.897
37	P2	0.053	0.823	0.100	0.502	0.008	0.975	-0.130	0.676
38	CP2	0.102	0.593	0.162	0.435	0.145	0.418	-0.050	0.964
39	P4	0.029	0.834	0.116	0.428	0.098	0.963	-0.094	0.736
40	P10	-0.080	0.907	-0.022	0.873	0.157	0.670	-0.041	0.998
41	T6	0.034	0.870	0.122	0.484	0.142	0.422	-0.060	0.917
42	P6	-0.022	0.962	0.051	0.708	0.190	0.615	0.006	0.994
43	CP6	0.026	0.991	0.114	0.470	0.179	0.798	0.024	0.934
44	A2	-0.137	0.733	-0.090	0.844	0.183	0.480	0.039	0.878
45	TP8	-0.013	0.952	0.044	0.747	0.162	0.796	0.028	0.912
46	C6	0.049	0.840	0.104	0.482	0.151	0.785	0.002	0.989
47	C4	0.029	0.967	0.094	0.578	0.133	0.786	-0.020	0.986
48	C2	-0.051	0.846	0.032	0.881	0.040	0.848	-0.094	0.839
49	T4	-0.019	0.888	-0.016	0.979	0.163	0.574	-0.144	0.928
50	FC4	0.115	0.519	0.136	0.359	0.037	0.924	-0.129	0.832
51	FC2	-0.025	0.923	-0.004	0.979	-0.006	0.986	-0.115	0.988
52	FT8	0.034	0.805	0.025	0.926	-0.009	0.986	-0.151	0.970
53	FC6	0.095	0.575	0.105	0.521	-0.004	0.974	-0.190	0.793
54	F8	-0.016	0.907	0.011	0.938	0.045	0.926	-0.144	0.854
55	F6	0.009	0.948	0.046	0.797	-0.043	0.947	-0.225	0.742
56	F4	-0.055	0.963	0.083	0.586	-0.048	0.894	-0.185	0.607
57	Cz	-0.030	0.896	0.006	0.967	0.102	0.567	-0.069	0.891

Supplementary Table S11. Correlation analysis result of alpha relative power and LF-HRV.

		Group 1. Depressive disorder				Group 2. Anxiety disorder			
Electrodes		Resting		Task		Resting		Task	
No.	Name	r	p (FDR)	r	p (FDR)	r	p (FDR)	r	p (FDR)
01	AF4	0.140	0.491	-0.093	0.805	-0.094	0.943	-0.180	0.845
02	F2	0.143	0.396	-0.053	0.906	-0.082	0.881	-0.187	0.915
03	FCz	0.050	0.931	-0.050	0.848	-0.056	0.860	-0.175	0.968
04	Fp2	0.136	0.506	-0.116	0.925	-0.174	0.844	-0.204	0.974
05	Fz	0.119	0.619	-0.094	0.994	-0.116	0.946	-0.193	0.866
06	FC1	0.077	0.671	-0.059	0.917	-0.006	0.965	-0.143	0.970
07	AFz	0.172	0.443	-0.155	0.685	-0.100	0.954	-0.232	0.787
08	F1	0.142	0.516	-0.079	0.942	-0.118	0.868	-0.231	0.817
09	Fp1	0.154	0.624	-0.157	0.833	-0.127	0.924	-0.223	0.585
10	AF3	0.084	0.697	-0.152	0.571	-0.118	0.899	-0.222	0.808
11	F3	0.069	0.727	-0.055	0.925	-0.071	0.913	-0.143	0.933
12	F5	0.077	0.675	-0.087	0.747	-0.098	0.727	-0.212	0.711
13	FC5	0.104	0.525	-0.026	0.936	-0.032	0.817	-0.164	0.743
14	FC3	0.120	0.446	-0.080	0.774	-0.005	0.973	-0.203	0.603
15	C1	-0.017	0.902	-0.176	0.361	0.048	0.733	-0.172	0.833
16	F7	-0.010	0.943	-0.070	0.833	-0.039	0.778	-0.142	0.796
17	FT7	0.097	0.564	-0.061	0.853	-0.024	0.863	-0.182	0.821
18	C3	0.034	0.806	-0.167	0.473	-0.010	0.945	-0.171	0.933
19	CP1	-0.023	0.867	-0.212	0.369	0.016	0.997	-0.126	0.975
20	C5	0.148	0.518	-0.110	0.612	0.031	0.822	-0.179	0.773
21	T3	0.023	0.979	-0.067	0.804	0.003	0.982	-0.144	0.940
22	TP7	0.172	0.423	-0.101	0.732	-0.006	0.965	-0.169	0.999
23	CP5	0.163	0.418	-0.109	0.774	0.014	0.918	-0.136	0.931
24	P5	0.143	0.534	-0.085	0.837	-0.047	0.867	-0.150	0.886
25	P3	0.117	0.507	-0.068	0.862	-0.070	0.930	-0.207	0.985
26	A1	0.098	0.850	-0.078	0.865	0.047	0.798	-0.098	0.892
27	T5	0.148	0.669	-0.016	0.977	-0.037	0.788	-0.146	0.947
28	P1	0.088	0.613	-0.050	0.898	-0.069	0.879	-0.232	0.802
29	P9	0.163	0.656	-0.041	0.905	-0.032	0.888	-0.178	0.865

30	PO3	0.049	0.908	-0.077	0.930	-0.050	0.804	-0.173	0.840
31	Pz	0.038	0.779	-0.096	0.893	-0.013	0.925	-0.136	0.849
32	O1	0.166	0.412	0.069	0.725	-0.048	0.792	-0.186	0.835
33	POz	0.025	0.856	-0.073	0.831	-0.021	0.879	-0.165	0.899
34	Oz	0.105	0.574	0.036	0.793	-0.051	0.774	-0.193	0.773
35	PO4	0.076	0.627	-0.046	0.898	-0.024	0.861	-0.162	0.799
36	O2	0.158	0.527	0.078	0.671	-0.058	0.725	-0.205	0.874
37	P2	0.153	0.460	0.013	0.925	-0.079	0.833	-0.209	0.708
38	CP2	0.192	0.406	0.010	0.993	0.015	0.914	-0.186	0.696
39	P4	0.171	0.426	-0.010	0.942	-0.045	0.851	-0.196	0.775
40	P10	0.080	0.942	-0.080	0.895	0.034	0.874	-0.142	0.916
41	T6	0.141	0.650	-0.003	0.980	0.029	0.891	-0.150	0.870
42	P6	0.101	0.778	-0.096	0.863	0.018	0.898	-0.176	0.722
43	CP6	0.129	0.629	-0.078	0.812	-0.017	0.903	-0.141	0.830
44	A2	-0.032	0.943	-0.185	0.372	0.064	0.701	-0.084	0.789
45	TP8	0.033	0.864	-0.146	0.497	0.058	0.799	-0.081	0.974
46	C6	0.110	0.725	-0.135	0.523	0.044	0.753	-0.040	0.937
47	C4	0.131	0.620	-0.075	0.900	0.015	0.915	-0.100	0.922
48	C2	0.092	0.724	-0.084	0.919	-0.010	0.943	-0.186	0.842
49	T4	-0.062	0.928	-0.155	0.473	-0.010	0.944	-0.131	0.680
50	FC4	0.172	0.332	-0.053	0.904	0.007	0.959	-0.154	0.814
51	FC2	0.050	0.773	-0.063	0.882	-0.019	0.891	-0.142	0.865
52	FT8	0.079	0.665	-0.109	0.702	-0.064	0.996	-0.124	0.889
53	FC6	0.122	0.479	-0.069	0.801	-0.069	0.977	-0.180	0.728
54	F8	-0.056	0.931	-0.127	0.725	-0.042	0.944	-0.096	0.937
55	F6	0.095	0.527	-0.076	0.798	-0.076	0.950	-0.140	0.879
56	F4	0.027	0.842	-0.091	0.912	-0.086	0.932	-0.096	0.933
57	Cz	-0.019	0.966	-0.155	0.470	-0.033	0.882	-0.143	0.783

Supplementary Table S12. Correlation analysis result of beta absolute power and LF-HRV.

Electrodes		Group 1. Depressive disorder				Group 2. Anxiety disorder			
		Resting		Task		Resting		Task	
No.	Name	r	p (FDR)	r	p (FDR)	r	p (FDR)	r	p (FDR)
01	AF4	-0.249	0.541	0.081	0.654	0.002	0.988	-0.258	0.406
02	F2	-0.219	0.550	0.093	0.619	-0.078	0.713	-0.215	0.607
03	FCz	-0.014	0.917	0.072	0.646	0.117	0.645	-0.008	0.979
04	Fp2	-0.167	0.786	0.084	0.699	-0.062	0.774	-0.108	0.776
05	Fz	-0.180	0.746	0.036	0.794	-0.159	0.547	-0.171	0.562
06	FC1	-0.203	0.756	0.028	0.929	0.203	0.485	0.069	0.863
07	AFz	-0.230	0.466	0.072	0.676	-0.036	0.928	-0.231	0.401
08	F1	-0.151	0.934	-0.029	0.848	0.042	0.924	-0.192	0.814
09	Fp1	-0.056	0.894	-0.006	0.966	-0.050	0.864	-0.132	0.736
10	AF3	-0.164	0.933	-0.018	0.894	-0.012	0.979	-0.267	0.319
11	F3	-0.137	0.931	0.021	0.953	-0.042	0.905	-0.122	0.853
12	F5	-0.222	0.833	0.011	0.938	0.089	0.916	-0.174	0.804
13	FC5	-0.221	0.733	0.067	0.675	0.083	0.818	-0.114	0.842
14	FC3	-0.265	0.607	0.014	0.916	0.116	0.778	0.005	0.969
15	C1	-0.249	0.612	-0.018	0.969	0.111	0.614	0.033	0.969
16	F7	-0.062	0.904	-0.045	0.895	0.064	0.841	-0.144	0.828
17	FT7	-0.248	0.754	0.035	0.894	0.067	0.820	-0.115	0.809
18	C3	-0.307	0.279	-0.075	0.950	0.135	0.860	-0.017	0.998
19	CP1	-0.264	0.517	-0.065	0.991	0.158	0.620	0.053	0.939
20	C5	-0.233	0.659	-0.003	0.980	0.116	0.937	0.027	0.923
21	T3	-0.032	0.841	-0.091	0.809	0.071	0.794	-0.109	0.936
22	TP7	-0.222	0.569	-0.057	0.998	0.085	0.853	-0.022	0.984
23	CP5	-0.237	0.425	-0.025	0.923	0.107	0.873	0.041	0.907
24	P5	-0.192	0.875	-0.002	0.989	0.127	0.759	0.036	0.982
25	P3	-0.209	0.581	0.004	0.975	0.130	0.754	0.034	0.873
26	A1	-0.202	0.446	-0.088	0.858	0.075	0.770	-0.086	0.981
27	T5	-0.206	0.655	-0.119	0.829	0.073	0.707	-0.052	0.977
28	P1	-0.169	0.739	-0.005	0.979	0.038	0.786	-0.053	0.968
29	P9	-0.102	0.969	-0.045	0.995	0.087	0.731	0.020	0.978

30	PO3	-0.232	0.460	0.026	0.921	0.141	0.638	-0.039	0.845
31	Pz	-0.264	0.646	-0.050	0.837	0.197	0.535	-0.047	0.991
32	O1	-0.118	0.980	-0.037	0.787	0.141	0.719	0.061	0.769
33	POz	-0.262	0.567	-0.040	0.850	0.188	0.687	-0.068	0.996
34	Oz	-0.139	0.973	-0.100	0.917	0.183	0.480	-0.001	0.998
35	PO4	-0.130	0.931	-0.003	0.989	0.156	0.700	0.006	0.985
36	O2	-0.124	0.875	0.010	0.940	0.217	0.381	0.001	0.996
37	P2	-0.100	0.850	0.085	0.995	0.137	0.873	-0.003	0.998
38	CP2	-0.060	0.915	0.088	0.815	0.176	0.472	0.028	1.000
39	P4	-0.185	0.629	0.086	0.573	0.161	0.837	-0.002	0.989
40	P10	-0.204	0.960	-0.062	0.982	0.241	0.670	0.060	0.983
41	T6	-0.122	0.862	0.104	0.484	0.186	0.653	0.037	0.810
42	P6	-0.153	0.960	0.047	0.793	0.220	0.690	0.090	0.982
43	CP6	-0.115	0.886	0.065	0.782	0.216	0.774	0.071	0.962
44	A2	-0.177	0.895	-0.096	0.668	0.256	0.361	0.107	0.980
45	TP8	-0.098	0.991	0.029	0.832	0.183	0.852	0.041	0.968
46	C6	-0.058	0.964	0.086	0.798	0.153	0.876	0.005	0.997
47	C4	-0.101	0.907	0.064	0.714	0.179	0.874	0.059	0.922
48	C2	-0.136	0.954	-0.037	0.938	0.139	0.718	0.018	0.971
49	T4	-0.014	0.954	-0.032	0.895	0.099	0.647	-0.088	0.899
50	FC4	-0.031	0.999	0.118	0.627	0.109	0.947	-0.030	0.994
51	FC2	-0.073	0.890	-0.035	0.980	0.078	0.921	-0.041	0.935
52	FT8	-0.131	0.996	0.013	0.928	0.112	0.910	-0.082	0.978
53	FC6	-0.114	0.953	0.083	0.611	0.091	0.849	-0.092	0.933
54	F8	-0.066	0.998	0.038	0.789	-0.031	0.950	-0.112	0.964
55	F6	-0.188	0.793	0.027	0.943	0.028	0.912	-0.170	0.977
56	F4	-0.180	0.942	0.086	0.641	-0.093	0.727	-0.162	0.712
57	Cz	-0.068	0.943	-0.013	0.942	0.152	0.602	-0.019	0.968

Supplementary Table S13. Correlation analysis result of beta relative power and LF-HRV.

		Group 1. Depressive disorder				Group 2. Anxiety disorder			
Electrodes		Resting		Task		Resting		Task	
No.	Name	r	p (FDR)	r	p (FDR)	r	p (FDR)	r	p (FDR)
01	AF4	-0.111	0.647	-0.346	**0.039***	-0.157	0.862	-0.085	0.787
02	F2	-0.111	0.622	-0.364	**0.025***	-0.146	0.790	-0.075	0.893
03	FCz	-0.076	0.628	-0.280	0.190	-0.042	0.960	-0.067	0.924
04	Fp2	-0.064	0.834	-0.275	0.174	-0.249	0.650	0.083	0.703
05	Fz	-0.101	0.703	-0.256	0.247	-0.195	0.626	-0.143	0.770
06	FC1	-0.070	0.764	-0.231	0.265	-0.043	0.991	-0.052	0.939
07	AFz	-0.123	0.475	-0.398	**0.010***	-0.160	0.762	-0.103	0.762
08	F1	-0.121	0.606	-0.344	**0.034***	-0.123	0.818	-0.063	0.973
09	Fp1	-0.081	0.655	-0.315	0.064	-0.213	0.730	-0.002	0.989
10	AF3	-0.135	0.536	-0.399	**0.010***	-0.192	0.752	-0.092	0.843
11	F3	-0.148	0.648	-0.257	0.214	-0.212	0.658	-0.026	0.930
12	F5	-0.164	0.420	-0.305	0.072	-0.146	0.865	-0.074	0.946
13	FC5	-0.157	0.460	-0.285	0.114	-0.126	0.753	-0.043	0.942
14	FC3	-0.179	0.345	-0.321	0.070	-0.095	0.998	-0.067	0.966
15	C1	-0.131	0.526	-0.266	0.178	-0.043	0.962	-0.023	0.909
16	F7	-0.213	0.277	-0.279	0.162	-0.166	0.763	-0.092	0.834
17	FT7	-0.202	0.252	-0.267	0.151	-0.138	0.873	-0.073	0.972
18	C3	-0.203	0.263	-0.329	**0.049***	-0.073	0.969	-0.005	0.972
19	CP1	-0.130	0.550	-0.268	0.218	-0.051	0.789	-0.049	0.961
20	C5	-0.257	0.122	-0.347	**0.028***	-0.089	0.983	-0.045	0.985
21	T3	-0.188	0.417	-0.188	0.535	-0.173	0.581	-0.175	0.735
22	TP7	-0.232	0.164	-0.360	**0.028***	-0.139	0.831	-0.080	0.920
23	CP5	-0.241	0.165	-0.386	**0.021***	-0.138	0.933	-0.073	0.935
24	P5	-0.195	0.215	-0.395	**0.018***	-0.183	0.740	-0.116	0.777
25	P3	-0.186	0.255	-0.350	**0.048***	-0.128	0.790	-0.068	0.963
26	A1	-0.195	0.225	-0.335	**0.046***	-0.154	0.953	-0.124	0.891
27	T5	-0.152	0.382	-0.364	0.070	-0.200	0.844	-0.136	0.863
28	P1	-0.159	0.348	-0.318	0.080	-0.087	0.939	-0.104	0.842
29	P9	-0.163	0.333	-0.348	0.054	-0.186	0.778	-0.098	0.893

30	PO3	-0.160	0.346	-0.331	0.057	-0.127	0.857	-0.111	0.966
31	Pz	-0.190	0.234	-0.329	0.053	-0.115	0.849	-0.089	0.971
32	O1	-0.189	0.301	-0.388	**0.025***	-0.152	0.844	-0.072	0.930
33	POz	-0.155	0.366	-0.354	**0.035***	-0.163	0.741	-0.120	0.863
34	Oz	-0.137	0.366	-0.358	**0.042***	-0.178	0.837	-0.092	0.939
35	PO4	-0.173	0.291	-0.372	**0.023***	-0.164	0.631	-0.109	0.925
36	O2	-0.160	0.345	-0.344	**0.039***	-0.155	0.868	-0.107	0.979
37	P2	-0.160	0.344	-0.369	**0.029***	-0.124	0.952	-0.066	0.955
38	CP2	-0.133	0.476	-0.313	0.052	-0.092	0.896	-0.091	0.957
39	P4	-0.150	0.438	-0.347	**0.041***	-0.128	0.848	-0.065	0.977
40	P10	-0.029	0.903	-0.271	0.170	-0.131	0.814	-0.080	0.955
41	T6	-0.082	0.794	-0.279	0.128	-0.172	0.901	-0.089	0.977
42	P6	-0.084	0.776	-0.301	0.103	-0.164	0.759	-0.106	0.883
43	CP6	-0.085	0.694	-0.289	0.110	-0.141	0.715	-0.099	0.912
44	A2	-0.024	0.915	-0.233	0.210	-0.156	0.961	-0.128	0.944
45	TP8	-0.053	0.909	-0.188	0.338	-0.132	0.800	-0.103	0.852
46	C6	-0.069	0.796	-0.235	0.190	-0.134	0.833	-0.090	0.943
47	C4	-0.071	0.786	-0.173	0.330	-0.121	0.962	-0.047	0.983
48	C2	-0.030	0.825	-0.187	0.297	-0.094	0.970	-0.093	0.902
49	T4	-0.153	0.508	-0.316	0.085	-0.277	0.225	0.005	0.986
50	FC4	-0.074	0.765	-0.204	0.243	-0.111	0.970	-0.090	0.956
51	FC2	-0.006	0.964	-0.137	0.458	-0.111	0.873	-0.133	0.789
52	FT8	-0.128	0.500	-0.184	0.325	-0.085	0.901	-0.072	0.935
53	FC6	-0.123	0.558	-0.231	0.188	-0.118	0.851	-0.069	0.974
54	F8	-0.237	0.187	-0.247	0.330	-0.190	0.862	-0.006	0.967
55	F6	-0.126	0.602	-0.243	0.230	-0.122	0.887	-0.067	0.966
56	F4	-0.111	0.638	-0.209	0.531	-0.175	0.784	-0.045	0.975
57	Cz	-0.089	0.557	-0.273	0.123	-0.088	0.789	-0.112	0.888

Supplementary Table S14. Correlation analysis result of gamma absolute power and LF-HRV.

Electrodes		Group 1. Depressive disorder				Group 2. Anxiety disorder			
		Resting		Task		Resting		Task	
No.	Name	r	p (FDR)	r	p (FDR)	r	p (FDR)	r	p (FDR)
01	AF4	-0.245	0.481	0.216	0.239	-0.051	0.830	-0.262	0.318
02	F2	-0.234	0.446	0.207	0.227	-0.167	0.592	-0.295	0.188
03	FCz	-0.077	0.776	0.076	0.623	-0.035	0.869	-0.113	0.885
04	Fp2	-0.189	0.629	0.093	0.587	0.062	0.924	-0.111	0.787
05	Fz	-0.198	0.391	0.053	0.697	-0.125	0.604	-0.127	0.787
06	FC1	-0.278	0.366	0.046	0.868	0.114	0.664	-0.040	0.915
07	AFz	-0.226	0.366	0.201	0.256	-0.016	0.985	-0.198	0.380
08	F1	-0.200	0.455	-0.008	0.951	0.020	0.884	-0.243	0.515
09	Fp1	-0.006	0.964	0.000	0.998	0.063	0.889	-0.123	0.760
10	AF3	-0.137	0.683	0.086	0.573	-0.022	0.898	-0.261	0.371
11	F3	-0.145	0.720	0.021	0.944	-0.030	0.978	-0.130	0.785
12	F5	-0.221	0.404	0.053	0.794	0.095	0.920	-0.226	0.498
13	FC5	-0.241	0.173	0.057	0.797	-0.018	0.962	-0.296	0.251
14	FC3	-0.290	0.204	0.057	0.756	-0.025	0.976	-0.137	0.840
15	C1	-0.273	0.285	0.005	0.971	0.097	0.700	-0.062	0.940
16	F7	-0.068	0.891	0.032	0.991	0.144	0.775	-0.152	0.839
17	FT7	-0.268	0.200	0.085	0.804	-0.010	0.941	-0.224	0.725
18	C3	-0.313	0.149	-0.020	0.881	-0.044	0.889	-0.191	0.559
19	CP1	-0.296	0.213	-0.010	0.941	0.006	0.965	-0.087	0.963
20	C5	-0.252	0.262	0.055	0.748	-0.089	0.755	-0.110	0.865
21	T3	-0.120	0.493	-0.096	0.627	0.024	0.994	-0.144	0.908
22	TP7	-0.264	0.214	-0.034	0.991	-0.085	0.829	-0.176	0.621
23	CP5	-0.271	0.186	0.036	0.793	-0.064	0.864	-0.138	0.592
24	P5	-0.264	0.213	0.043	0.819	-0.010	0.942	-0.129	0.982
25	P3	-0.259	0.235	0.015	0.938	0.013	0.925	-0.128	0.770
26	A1	-0.239	0.248	-0.084	0.881	-0.053	0.949	-0.242	0.393
27	T5	-0.325	0.066	-0.240	0.490	-0.051	0.846	-0.171	0.849
28	P1	-0.256	0.197	0.013	0.927	0.002	0.990	-0.107	0.938
29	P9	-0.198	0.593	-0.113	0.921	-0.006	0.965	-0.086	0.999

30	PO3	-0.267	0.250	0.077	0.743	0.177	0.723	-0.090	0.865
31	Pz	-0.270	0.295	-0.012	0.928	0.166	0.773	-0.090	0.836
32	O1	-0.258	0.239	-0.164	0.845	0.196	0.593	-0.016	0.910
33	POz	-0.287	0.240	-0.053	0.903	0.213	0.565	-0.136	0.929
34	Oz	-0.279	0.250	-0.248	0.693	0.236	0.549	-0.082	0.971
35	PO4	-0.235	0.300	-0.010	0.997	0.210	0.339	-0.085	0.859
36	O2	-0.241	0.346	-0.027	0.841	0.265	0.296	-0.076	0.948
37	P2	-0.211	0.383	0.117	0.858	0.072	0.952	-0.107	0.875
38	CP2	-0.200	0.370	0.115	0.780	0.140	0.553	-0.048	0.999
39	P4	-0.281	0.133	0.085	0.595	0.038	0.949	-0.156	0.978
40	P10	-0.288	0.334	-0.199	0.859	0.013	0.925	-0.167	0.597
41	T6	-0.189	0.301	0.091	0.654	0.078	0.906	-0.132	0.796
42	P6	-0.303	0.213	0.034	0.870	0.144	0.971	-0.094	0.914
43	CP6	-0.257	0.112	0.043	0.973	0.122	0.928	-0.091	0.985
44	A2	-0.125	0.606	-0.041	0.908	0.073	0.999	-0.093	0.932
45	TP8	-0.249	0.176	0.023	0.936	-0.007	0.961	-0.131	0.867
46	C6	-0.169	0.459	0.190	0.297	-0.022	0.946	-0.170	0.934
47	C4	-0.215	0.460	0.099	0.673	-0.013	0.928	-0.076	0.975
48	C2	-0.285	0.178	-0.079	0.928	0.038	0.870	-0.070	0.850
49	T4	0.061	0.946	0.003	0.980	0.134	0.678	-0.173	0.829
50	FC4	-0.128	0.518	0.211	0.308	-0.014	0.969	-0.123	0.954
51	FC2	-0.123	0.678	-0.079	0.881	-0.028	0.948	-0.140	0.955
52	FT8	-0.225	0.479	0.081	0.886	-0.078	0.801	-0.199	0.679
53	FC6	-0.180	0.479	0.175	0.429	-0.046	0.856	-0.169	0.862
54	F8	-0.132	0.535	0.112	0.737	-0.093	0.729	-0.150	0.913
55	F6	-0.220	0.514	0.135	0.675	-0.097	0.789	-0.237	0.602
56	F4	-0.191	0.605	0.179	0.345	-0.127	0.670	-0.147	0.811
57	Cz	-0.158	0.532	-0.035	0.826	0.047	0.842	-0.084	0.829

Supplementary Table S15. Correlation analysis result of gamma relative power and LF-HRV.

Electrodes		Group 1. Depressive disorder				Group 2. Anxiety disorder			
		Resting		Task		Resting		Task	
No.	Name	r	p (FDR)	r	p (FDR)	r	p (FDR)	r	p (FDR)
01	AF4	-0.149	0.440	-0.114	0.582	-0.122	0.932	-0.057	0.992
02	F2	-0.165	0.511	-0.146	0.449	-0.101	0.838	0.015	0.916
03	FCz	-0.177	0.383	-0.188	0.404	-0.089	0.994	-0.032	0.837
04	Fp2	-0.053	0.701	-0.143	0.848	0.001	0.994	-0.072	0.884
05	Fz	-0.087	0.835	-0.180	0.528	-0.032	0.915	-0.054	0.945
06	FC1	-0.196	0.340	-0.181	0.395	-0.068	0.949	-0.012	0.962
07	AFz	-0.115	0.677	-0.174	0.387	-0.069	0.928	-0.022	0.999
08	F1	-0.157	0.641	-0.243	0.154	-0.049	0.999	-0.025	0.996
09	Fp1	-0.039	0.941	-0.247	0.430	-0.006	0.994	-0.090	0.991
10	AF3	-0.070	0.876	-0.196	0.295	-0.116	0.934	-0.072	0.956
11	F3	-0.157	0.643	-0.179	0.472	-0.105	0.852	-0.178	0.675
12	F5	-0.162	0.603	-0.147	0.455	-0.083	0.998	-0.077	0.987
13	FC5	-0.209	0.314	-0.191	0.343	-0.112	0.990	-0.101	0.876
14	FC3	-0.227	0.270	-0.182	0.334	-0.138	0.938	-0.022	0.991
15	C1	-0.197	0.280	-0.189	0.354	-0.061	0.967	-0.001	0.997
16	F7	-0.170	0.713	-0.151	0.489	-0.087	0.628	-0.200	0.633
17	FT7	-0.245	0.256	-0.210	0.263	-0.095	0.887	-0.081	0.812
18	C3	-0.229	0.283	-0.201	0.298	-0.124	0.735	-0.060	0.979
19	CP1	-0.198	0.300	-0.184	0.361	-0.143	0.870	-0.101	0.870
20	C5	-0.289	0.124	-0.210	0.248	-0.159	0.763	-0.049	0.982
21	T3	-0.230	0.528	-0.208	0.350	-0.133	0.988	-0.301	0.166
22	TP7	-0.282	0.141	-0.219	0.193	-0.162	0.925	-0.088	0.788
23	CP5	-0.270	0.192	-0.215	0.243	-0.165	0.876	-0.070	0.909
24	P5	-0.253	0.259	-0.276	0.148	-0.173	0.819	-0.096	0.897
25	P3	-0.228	0.397	-0.261	0.134	-0.119	0.973	-0.045	0.986
26	A1	-0.233	0.256	-0.216	0.217	-0.173	0.802	-0.217	0.496
27	T5	-0.262	0.222	-0.316	0.077	-0.211	0.625	-0.084	0.978
28	P1	-0.231	0.305	-0.249	0.166	-0.055	0.993	-0.048	0.956
29	P9	-0.251	0.200	-0.306	0.071	-0.153	0.770	-0.045	0.984

30	PO3	-0.246	0.293	-0.247	0.186	-0.009	0.978	-0.042	0.918
31	Pz	-0.216	0.435	-0.201	0.525	-0.076	0.887	-0.080	0.871
32	O1	-0.240	0.323	-0.353	0.083	-0.003	0.985	0.026	0.851
33	POz	-0.214	0.448	-0.241	0.312	-0.020	0.902	-0.025	0.962
34	Oz	-0.215	0.451	-0.307	0.136	-0.017	0.931	0.006	0.964
35	PO4	-0.218	0.387	-0.242	0.301	-0.011	0.939	-0.016	0.911
36	O2	-0.207	0.542	-0.252	0.259	0.033	0.959	0.043	0.759
37	P2	-0.209	0.479	-0.226	0.238	-0.016	0.996	0.044	0.766
38	CP2	-0.252	0.265	-0.218	0.231	-0.030	0.993	0.042	0.901
39	P4	-0.253	0.169	-0.209	0.256	-0.125	0.937	-0.028	0.841
40	P10	-0.146	0.682	-0.259	0.175	-0.235	0.376	-0.150	0.727
41	T6	-0.163	0.372	-0.174	0.339	-0.207	0.395	-0.087	0.803
42	P6	-0.216	0.301	-0.218	0.231	-0.157	0.747	-0.064	0.859
43	CP6	-0.244	0.152	-0.249	0.155	-0.121	0.971	-0.068	0.812
44	A2	-0.037	0.785	-0.139	0.498	-0.187	0.468	-0.157	0.497
45	TP8	-0.236	0.174	-0.160	0.441	-0.189	0.720	-0.086	0.897
46	C6	-0.240	0.239	-0.107	0.609	-0.167	0.740	-0.143	0.738
47	C4	-0.269	0.196	-0.151	0.483	-0.146	0.829	-0.017	0.979
48	C2	-0.241	0.237	-0.202	0.280	-0.084	0.911	0.004	0.980
49	T4	-0.093	0.616	-0.146	0.467	-0.121	0.897	-0.238	0.852
50	FC4	-0.224	0.381	-0.140	0.440	-0.126	0.796	-0.011	0.997
51	FC2	-0.162	0.570	-0.186	0.368	-0.089	0.956	0.009	0.997
52	FT8	-0.237	0.276	-0.096	0.627	-0.160	0.810	-0.086	0.981
53	FC6	-0.210	0.408	-0.150	0.439	-0.149	0.850	-0.029	0.991
54	F8	-0.196	0.476	-0.067	0.780	-0.212	0.360	-0.232	0.419
55	F6	-0.192	0.450	-0.072	0.707	-0.151	0.918	-0.058	0.987
56	F4	-0.082	0.746	-0.119	0.619	-0.168	0.797	-0.155	0.761
57	Cz	-0.121	0.559	-0.253	0.195	-0.077	0.985	-0.187	0.457

Supplementary Table S16. Correlation analysis result of delta absolute power and HF-HRV.

		Group 1. Depressive disorder				Group 2. Anxiety disorder			
Electrodes		Resting		Task		Resting		Task	
No.	Name	r	p (FDR)	r	p (FDR)	r	p (FDR)	r	p (FDR)
01	AF4	-0.112	0.643	0.420	**0.006***	-0.072	0.852	-0.157	0.457
02	F2	-0.074	0.696	0.487	**0.001***	-0.166	0.746	-0.144	0.708
03	FCz	0.099	0.608	0.34	**0.044***	-0.066	0.941	0.047	0.957
04	Fp2	-0.056	0.807	0.408	**0.022***	-0.071	0.846	-0.140	0.653
05	Fz	0.000	0.999	0.467	**0.004***	-0.092	0.920	-0.121	0.684
06	FC1	-0.119	0.696	0.195	0.389	-0.013	0.950	0.024	0.947
07	AFz	-0.051	0.767	0.479	**0.001***	-0.058	0.794	0.003	0.982
08	F1	-0.028	0.948	0.371	**0.021***	-0.008	0.956	-0.053	0.869
09	Fp1	0.041	0.91	0.369	**0.022***	-0.021	0.956	-0.14	0.575
10	AF3	-0.05	0.989	0.410	**0.006***	-0.091	0.843	-0.088	0.761
11	F3	0.001	0.995	0.345	**0.033***	-0.11	0.815	-0.167	0.690
12	F5	-0.044	0.887	0.32	**0.042***	0.027	0.918	-0.109	0.704
13	FC5	0.009	0.977	0.361	**0.017***	0.034	0.915	-0.109	0.776
14	FC3	-0.064	0.808	0.331	**0.033***	-0.009	0.984	0.000	1.000
15	C1	-0.099	0.759	0.261	0.136	0.033	0.878	-0.022	0.983
16	F7	0.046	0.873	0.233	0.217	0.081	0.971	-0.159	0.707
17	FT7	-0.006	0.968	0.286	0.084	0.059	0.938	-0.089	0.885
18	C3	-0.100	0.863	0.261	0.135	-0.042	0.812	-0.066	0.976
19	CP1	-0.091	0.722	0.255	0.150	0.019	0.893	-0.018	0.946
20	C5	-0.029	0.865	0.288	0.081	-0.083	0.862	-0.032	0.986
21	T3	0.141	0.497	0.184	0.479	-0.101	0.783	-0.121	0.913
22	TP7	-0.047	0.789	0.277	0.100	-0.038	0.867	-0.054	0.871
23	CP5	-0.047	0.951	0.307	0.055	-0.055	0.854	-0.046	0.969
24	P5	-0.022	0.992	0.347	**0.029***	0.023	0.938	-0.03	0.927
25	P3	-0.035	0.994	0.324	**0.039***	0.003	0.982	-0.054	0.957
26	A1	0.004	0.974	0.251	0.269	-0.01	0.975	-0.068	0.946
27	T5	-0.05	0.957	0.170	0.545	0.02	0.9	-0.058	0.920
28	P1	0.002	0.99	0.269	0.145	-0.06	0.869	-0.071	0.963
29	P9	0.048	0.795	0.341	0.059	0.057	0.809	0.026	0.986

30	PO3	-0.007	0.961	0.332	**0.040***	-0.112	0.782	-0.079	0.928
31	Pz	-0.002	0.988	0.278	0.123	-0.078	0.829	-0.105	0.9
32	O1	0.087	0.566	0.262	0.154	-0.006	0.982	0.055	0.98
33	POz	-0.022	0.923	0.298	0.084	-0.091	0.813	-0.104	0.94
34	Oz	0.016	0.918	0.127	0.629	-0.029	0.979	0.024	0.991
35	PO4	0.053	0.911	0.247	0.214	-0.097	0.687	-0.1	0.955
36	O2	-0.014	0.962	0.232	0.222	-0.058	0.797	0	0.997
37	P2	0.058	0.898	0.286	0.084	-0.142	0.606	-0.137	0.848
38	CP2	0.056	0.907	0.352	**0.020***	0.002	0.986	-0.047	0.98
39	P4	0.009	0.949	0.371	**0.013***	-0.055	0.802	-0.115	0.979
40	P10	-0.075	0.913	0.279	0.098	-0.031	0.943	-0.094	0.932
41	T6	0.021	0.94	0.397	**0.012***	0.06	0.785	-0.048	0.894
42	P6	-0.008	0.99	0.38	**0.012***	0.002	0.994	-0.03	0.997
43	CP6	0.041	0.932	0.359	**0.021***	0.013	0.974	-0.031	0.954
44	A2	0.013	0.938	0.34	**0.034***	0.117	0.975	-0.024	0.967
45	TP8	0.064	0.914	0.319	**0.043***	-0.035	0.968	-0.073	0.925
46	C6	0.025	0.993	0.361	**0.016***	-0.035	0.949	-0.103	0.978
47	C4	-0.012	0.999	0.298	0.067	-0.053	0.945	-0.114	0.997
48	C2	-0.071	0.747	0.204	0.338	-0.11	0.777	-0.084	0.91
49	T4	0.176	0.579	0.398	**0.020***	0.151	0.792	-0.125	0.85
50	FC4	0.016	0.905	0.345	**0.024***	-0.147	0.729	-0.145	0.871
51	FC2	-0.033	0.947	0.209	0.274	-0.181	0.668	-0.095	0.966
52	FT8	0.018	0.892	0.253	0.155	-0.004	0.977	-0.130	0.693
53	FC6	0.015	0.914	0.403	**0.006***	-0.081	0.918	-0.137	0.837
54	F8	0.073	0.698	0.325	**0.048***	0.110	0.966	-0.155	0.773
55	F6	-0.057	0.944	0.276	0.103	-0.06	0.969	-0.181	0.414
56	F4	-0.042	0.852	0.422	**0.004***	-0.023	0.989	-0.176	0.541
57	Cz	0.080	0.659	0.349	**0.036***	0.016	0.917	-0.100	0.945

Supplementary Table S17. Correlation analysis result of delta relative power and HF-HRV.

Electrodes		Group 1. Depressive disorder				Group 2. Anxiety disorder			
		Resting		Task		Resting		Task	
No.	Name	r	p (FDR)	r	p (FDR)	r	p (FDR)	r	p (FDR)
01	AF4	-0.151	0.831	0.169	0.462	0.027	0.948	0.006	0.963
02	F2	-0.138	0.848	0.169	0.463	-0.059	0.873	-0.026	0.981
03	FCz	-0.091	0.912	0.105	0.640	-0.212	0.406	0.056	0.966
04	Fp2	-0.119	0.96	0.286	0.142	-0.002	0.986	-0.025	0.96
05	Fz	-0.081	0.981	0.271	0.141	-0.027	0.968	0.072	0.984
06	FC1	-0.079	0.982	0.156	0.501	-0.21	0.345	0.001	0.993
07	AFz	-0.132	0.94	0.276	0.172	-0.043	0.898	0.094	0.966
08	F1	-0.112	0.865	0.208	0.280	-0.028	0.859	0.024	0.88
09	Fp1	-0.071	0.978	0.308	0.068	0.013	0.98	0.011	0.984
10	AF3	-0.055	0.931	0.248	0.171	0.061	0.862	0.056	0.891
11	F3	0.009	0.946	0.199	0.370	0.004	0.977	-0.031	0.961
12	F5	-0.029	0.899	0.16	0.389	0.042	0.889	-0.016	0.906
13	FC5	-0.044	0.875	0.136	0.460	-0.043	0.805	-0.024	0.948
14	FC3	-0.081	0.811	0.19	0.262	-0.158	0.299	0.007	0.962
15	C1	-0.044	0.858	0.195	0.323	-0.242	0.218	-0.02	0.885
16	F7	0.044	0.747	0.204	0.425	-0.083	0.748	-0.066	0.991
17	FT7	-0.023	0.869	0.128	0.547	-0.005	0.978	-0.008	0.956
18	C3	-0.012	0.928	0.262	0.166	-0.178	0.286	-0.04	0.944
19	CP1	-0.027	0.841	0.218	0.230	-0.212	0.261	-0.011	0.939
20	C5	-0.01	0.944	0.239	0.247	-0.183	0.318	-0.007	0.961
21	T3	-0.031	0.885	0.066	0.682	-0.269	0.263	0.015	0.983
22	TP7	-0.045	0.792	0.242	0.232	-0.159	0.326	-0.013	0.925
23	CP5	-0.037	0.904	0.279	0.12	-0.192	0.295	-0.053	0.872
24	P5	-0.054	0.985	0.268	0.149	-0.16	0.385	-0.03	0.831
25	P3	-0.061	0.95	0.214	0.247	-0.135	0.533	-0.028	0.838
26	A1	-0.016	0.957	0.231	0.28	-0.255	0.212	-0.012	0.974
27	T5	-0.085	0.92	0.16	0.39	-0.167	0.411	-0.003	0.983
28	P1	-0.06	0.999	0.119	0.555	-0.154	0.566	-0.007	0.962
29	P9	-0.092	0.911	0.209	0.257	-0.203	0.347	0.049	0.901

30	PO3	-0.066	0.902	0.141	0.434	-0.202	0.37	-0.005	0.989
31	Pz	0.01	0.948	0.166	0.322	-0.226	0.262	-0.015	0.954
32	O1	-0.106	0.994	0.011	0.995	-0.204	0.3	-0.007	0.959
33	POz	-0.025	0.933	0.156	0.364	-0.208	0.302	0.02	0.975
34	Oz	-0.079	0.971	0.022	0.943	-0.169	0.478	0.03	0.991
35	PO4	-0.02	0.945	0.114	0.581	-0.172	0.397	0.056	0.935
36	O2	-0.09	0.973	0.032	0.92	-0.194	0.394	0.053	0.948
37	P2	-0.073	0.92	0.108	0.556	-0.144	0.614	0.01	0.943
38	CP2	-0.083	0.941	0.129	0.56	-0.23	0.246	0.012	0.941
39	P4	-0.127	0.917	0.122	0.601	-0.149	0.527	-0.011	0.938
40	P10	-0.078	0.861	0.214	0.244	-0.216	0.379	-0.012	0.997
41	T6	-0.099	0.911	0.165	0.452	-0.191	0.394	-0.065	0.885
42	P6	-0.077	0.972	0.22	0.224	-0.217	0.298	-0.034	0.95
43	CP6	-0.097	0.93	0.19	0.316	-0.173	0.455	-0.016	0.911
44	A2	-0.043	0.883	0.18	0.299	-0.168	0.605	-0.008	0.999
45	TP8	-0.053	0.958	0.192	0.285	-0.161	0.456	-0.086	0.778
46	C6	-0.098	0.966	0.233	0.239	-0.127	0.49	-0.074	0.822
47	C4	-0.115	0.989	0.129	0.559	-0.175	0.353	-0.091	0.729
48	C2	-0.107	0.973	0.181	0.339	-0.183	0.4	0.01	0.963
49	T4	0.041	0.767	0.225	0.217	-0.074	0.771	-0.006	0.966
50	FC4	-0.166	0.722	0.126	0.524	-0.192	0.41	-0.052	0.853
51	FC2	-0.098	0.908	0.128	0.563	-0.219	0.366	-0.005	0.97
52	FT8	-0.069	0.872	0.146	0.504	0.028	0.983	-0.08	0.819
53	FC6	-0.135	0.956	0.149	0.444	-0.04	0.837	-0.06	0.807
54	F8	0.066	0.741	0.221	0.266	-0.056	0.894	-0.181	0.44
55	F6	-0.112	0.918	0.137	0.511	0.028	0.963	-0.077	0.838
56	F4	-0.012	0.929	0.242	0.236	-0.006	0.964	-0.052	0.854
57	Cz	-0.015	0.977	0.237	0.154	-0.231	0.311	0.011	0.953

Supplementary Table S18. Correlation analysis result of theta absolute power and HF-HRV.

		Group 1. Depressive disorder				Group 2. Anxiety disorder			
Electrodes		Resting		Task		Resting		Task	
No.	Name	r	p (FDR)	r	p (FDR)	r	p (FDR)	r	p (FDR)
01	AF4	-0.085	0.686	0.291	0.097	0.002	0.991	-0.063	1.000
02	F2	-0.024	0.858	0.295	0.117	-0.06	0.938	-0.061	0.996
03	FCz	0.149	0.508	0.247	0.227	0.062	0.922	0.041	0.916
04	Fp2	-0.024	0.91	0.292	0.189	0.071	0.866	-0.102	0.856
05	Fz	0.06	0.783	0.26	0.171	0.03	0.831	-0.106	0.803
06	FC1	-0.094	0.784	0.075	0.927	0.104	0.713	0.054	0.825
07	AFz	0.021	0.896	0.364	0.074	0.027	0.969	0.003	0.983
08	F1	0.02	0.883	0.204	0.287	0.051	0.884	0.024	0.863
09	Fp1	0.01	0.967	0.223	0.271	0.075	0.976	-0.107	0.839
10	AF3	-0.066	0.787	0.28	0.158	-0.031	0.973	-0.005	0.987
11	F3	0.002	0.986	0.199	0.304	0.014	0.917	-0.117	0.861
12	F5	-0.069	0.75	0.193	0.319	0.067	0.742	0.02	0.951
13	FC5	-0.002	0.989	0.246	0.2	0.078	0.808	0.024	0.921
14	FC3	-0.065	0.689	0.184	0.38	0.091	0.913	0.097	0.879
15	C1	-0.067	0.676	0.129	0.625	0.103	0.848	0.031	0.84
16	F7	0.058	0.925	0.099	0.573	0.147	0.946	-0.12	0.838
17	FT7	-0.012	0.987	0.2	0.26	0.09	0.776	0.008	0.997
18	C3	-0.084	0.874	0.12	0.548	0.092	0.945	0.054	0.922
19	CP1	-0.043	0.819	0.106	0.71	0.109	0.802	0.022	0.884
20	C5	-0.014	0.918	0.173	0.332	0.078	0.902	0.054	0.877
21	T3	0.183	0.367	0.087	0.811	0.119	0.599	-0.088	0.976
22	TP7	-0.013	0.927	0.149	0.461	0.108	0.855	0.022	0.943
23	CP5	-0.02	0.994	0.16	0.375	0.115	0.785	0.071	0.85
24	P5	0.001	0.993	0.198	0.361	0.15	0.682	0.076	0.839
25	P3	-0.015	0.984	0.161	0.434	0.129	0.822	0.073	0.912
26	A1	0.046	0.906	0.135	0.678	0.194	0.645	0.022	0.876
27	T5	0.005	0.971	0.084	0.779	0.121	0.726	0.023	0.919
28	P1	0.021	0.914	0.145	0.536	0.101	0.913	0.018	0.956
29	P9	0.095	0.636	0.205	0.399	0.174	0.541	0.093	0.77

30	PO3	-0.008	0.969	0.167	0.473	0.062	0.83	-0.017	0.902
31	Pz	-0.041	0.829	0.111	0.493	0.082	0.728	-0.08	0.973
32	O1	0.062	0.705	0.161	0.486	0.137	0.703	0.143	0.924
33	POz	-0.062	0.702	0.116	0.638	0.058	0.883	-0.03	0.957
34	Oz	-0.03	0.914	0.035	0.936	0.109	0.624	0.108	0.998
35	PO4	-0.012	0.928	0.077	0.747	0.055	0.882	-0.052	0.927
36	O2	-0.019	0.951	0.134	0.537	0.101	0.641	0.086	0.772
37	P2	0.039	0.988	0.111	0.542	0.028	0.992	-0.02	0.883
38	CP2	0.074	0.85	0.174	0.418	0.098	0.784	-0.015	0.977
39	P4	0.038	0.843	0.192	0.256	0.083	0.798	-0.002	0.99
40	P10	-0.046	0.835	0.091	0.661	0.136	0.534	0.027	0.848
41	T6	0.059	0.746	0.203	0.333	0.176	0.546	0.078	0.77
42	P6	0.021	0.941	0.161	0.398	0.126	0.592	0.07	0.785
43	CP6	0.074	0.87	0.185	0.322	0.112	0.681	0.052	0.996
44	A2	0.028	0.922	0.142	0.654	0.171	0.566	0.044	0.812
45	TP8	0.071	0.849	0.143	0.477	0.069	0.844	0.033	0.936
46	C6	0.06	0.99	0.169	0.358	0.052	0.9	-0.007	0.998
47	C4	0.03	0.991	0.192	0.339	0.053	0.887	-0.024	0.934
48	C2	-0.037	0.952	0.066	0.96	-0.002	0.989	-0.05	0.928
49	T4	0.155	0.655	0.223	0.258	0.207	0.431	-0.099	0.999
50	FC4	0.055	0.891	0.205	0.338	0.003	0.983	-0.044	0.948
51	FC2	0.002	0.994	0.091	0.962	-0.032	0.921	-0.067	0.834
52	FT8	0.046	0.958	0.129	0.635	0.028	0.912	0.003	0.986
53	FC6	0.052	0.881	0.258	0.216	0.014	0.987	-0.021	0.972
54	F8	0.11	0.672	0.194	0.331	0.159	0.831	-0.099	0.922
55	F6	-0.024	0.997	0.175	0.367	0.004	0.974	-0.032	0.927
56	F4	-0.022	0.871	0.216	0.229	0.084	0.99	-0.116	0.872
57	Cz	0.132	0.598	0.201	0.299	0.137	0.72	-0.081	0.939

Supplementary Table S19. Correlation analysis result of theta relative power and HF-HRV.

		Group 1. Depressive disorder				Group 2. Anxiety disorder			
Electrodes		Resting		Task		Resting		Task	
No.	Name	r	p (FDR)	r	p (FDR)	r	p (FDR)	r	p (FDR)
01	AF4	0.009	0.975	-0.038	0.923	0.033	0.882	0.216	0.333
02	F2	-0.013	0.941	-0.023	0.868	0.044	0.751	0.173	0.463
03	FCz	0.037	0.977	0.050	0.989	0.046	0.804	0.056	0.864
04	Fp2	0.017	1.000	-0.010	0.940	0.124	0.688	0.251	0.218
05	Fz	0.056	0.809	-0.015	0.911	0.098	0.626	0.189	0.555
06	FC1	-0.005	0.993	-0.029	0.954	0.045	0.837	0.056	0.878
07	AFz	0.095	0.956	-0.013	0.923	0.078	0.798	0.188	0.559
08	F1	0.012	0.998	-0.023	0.979	0.032	0.817	0.178	0.524
09	Fp1	-0.078	0.875	0.01	0.989	0.088	0.762	0.233	0.293
10	AF3	-0.07	0.983	-0.012	0.982	0.014	0.918	0.221	0.35
11	F3	-0.073	0.773	-0.134	0.527	0.024	0.866	0.167	0.492
12	F5	-0.082	0.964	-0.095	0.854	-0.015	0.912	0.181	0.497
13	FC5	-0.054	0.943	-0.086	0.869	0.003	0.983	0.149	0.497
14	FC3	-0.002	0.987	-0.065	0.951	0.05	0.862	0.129	0.51
15	C1	0.098	0.804	-0.004	0.98	0.004	0.976	0.053	0.983
16	F7	0.037	0.97	-0.132	0.48	0.044	0.887	0.106	0.643
17	FT7	0.008	0.955	-0.038	0.876	-0.008	0.99	0.125	0.681
18	C3	0.104	0.79	0.034	0.999	0.058	0.815	0.143	0.653
19	CP1	0.153	0.74	0.047	0.972	0.006	0.967	0.063	0.873
20	C5	0.074	0.696	0.029	0.984	0.068	0.808	0.127	0.669
21	T3	0.146	0.78	0.005	0.998	0.069	0.733	0.185	0.389
22	TP7	0.101	0.891	0.028	0.913	0.048	0.964	0.12	0.633
23	CP5	0.086	0.745	0.039	0.991	0.084	0.808	0.129	0.626
24	P5	0.039	0.946	0.025	0.996	0.079	0.821	0.121	0.62
25	P3	0.038	0.955	0	0.998	0.104	0.854	0.172	0.553
26	A1	0.099	0.703	0.034	0.873	0.077	0.794	0.154	0.621
27	T5	0.052	0.986	0.027	0.948	0.064	0.811	0.111	0.751
28	P1	-0.027	0.989	-0.021	0.974	0.096	0.797	0.188	0.455
29	P9	0.079	0.934	0.057	0.967	0.059	0.84	0.144	0.655

30	PO3	-0.063	0.973	-0.069	0.991	0.08	0.836	0.172	0.658
31	Pz	-0.053	0.986	-0.054	0.896	0.06	0.899	0.16	0.773
32	O1	-0.106	0.893	-0.016	0.997	0.065	0.811	0.17	0.638
33	POz	-0.074	0.993	-0.06	0.952	0.058	0.8	0.168	0.729
34	Oz	-0.088	0.858	-0.041	0.914	0.075	0.725	0.19	0.547
35	PO4	-0.09	0.797	-0.056	0.917	0.05	0.852	0.152	0.885
36	O2	-0.074	0.741	-0.128	0.928	0.094	0.753	0.193	0.527
37	P2	-0.067	0.902	-0.105	0.838	0.068	0.95	0.189	0.558
38	CP2	0.01	0.983	-0.062	0.987	-0.056	0.816	0.052	0.947
39	P4	-0.043	0.952	-0.124	0.91	0.074	0.858	0.187	0.427
40	P10	0.024	0.978	0.019	0.931	0.075	0.766	0.158	0.421
41	T6	-0.016	0.974	-0.129	0.854	0.053	0.833	0.151	0.46
42	P6	0.018	0.955	-0.114	0.895	0.052	0.922	0.161	0.4
43	CP6	0.028	0.839	-0.098	0.947	0.052	0.837	0.182	0.438
44	A2	0.046	0.989	0.017	0.962	0.041	0.847	0.076	0.731
45	TP8	0.062	0.923	-0.083	0.984	0.04	0.836	0.163	0.438
46	C6	0.068	0.933	-0.087	0.993	0.019	0.905	0.146	0.474
47	C4	0.058	0.927	-0.031	0.998	0.006	0.968	0.103	0.647
48	C2	0.076	0.93	-0.07	0.988	-0.018	0.997	0.061	0.857
49	T4	0.137	0.872	0.012	0.929	0.169	0.578	0.127	0.52
50	FC4	0	0.998	-0.061	0.994	0.015	0.995	0.141	0.501
51	FC2	0.009	0.996	-0.05	0.919	0.021	0.966	0.048	0.76
52	FT8	0.007	0.964	-0.124	0.93	0.019	0.951	0.222	0.349
53	FC6	-0.025	0.941	-0.079	0.896	0.018	0.902	0.196	0.44
54	F8	0.167	0.759	-0.039	0.941	0.094	0.814	0.181	0.414
55	F6	-0.006	0.99	-0.093	0.778	-0.003	0.98	0.218	0.362
56	F4	0.028	0.986	-0.14	0.612	0.103	0.854	0.218	0.323
57	Cz	0.111	0.975	0.01	0.941	0.028	0.939	0.106	0.724

Supplementary Table S20. Correlation analysis result of alpha absolute power and HF-HRV.

		Group 1. Depressive disorder				Group 2. Anxiety disorder			
Electrodes		Resting		Task		Resting		Task	
No.	Name	r	p (FDR)	r	p (FDR)	r	p (FDR)	r	p (FDR)
01	AF4	0.075	0.808	0.240	0.123	-0.036	0.975	-0.239	0.356
02	F2	0.147	0.598	0.26	0.087	-0.06	0.863	-0.206	0.437
03	FCz	0.200	0.361	0.237	0.171	0.073	0.867	-0.041	0.907
04	Fp2	0.114	0.722	0.178	0.311	0.004	0.978	-0.218	0.534
05	Fz	0.147	0.457	0.18	0.300	-0.011	0.969	-0.207	0.405
06	FC1	0.015	0.935	0.104	0.603	0.123	0.761	-0.025	0.955
07	AFz	0.144	0.538	0.239	0.124	-0.044	0.964	-0.217	0.376
08	F1	0.153	0.425	0.179	0.304	0.018	0.941	-0.166	0.600
09	Fp1	0.122	0.535	0.101	0.589	0.016	0.909	-0.231	0.36
10	AF3	0.115	0.577	0.178	0.309	-0.044	0.954	-0.234	0.288
11	F3	0.121	0.540	0.188	0.240	0.056	0.951	-0.198	0.589
12	F5	0.118	0.558	0.212	0.191	0.071	0.973	-0.142	0.793
13	FC5	0.165	0.414	0.284	0.069	0.113	0.813	-0.095	0.969
14	FC3	0.102	0.804	0.238	0.144	0.123	0.811	-0.024	0.942
15	C1	-0.018	0.942	0.119	0.683	0.189	0.523	0.014	0.985
16	F7	0.056	0.741	0.102	0.658	0.236	0.706	-0.153	0.8
17	FT7	0.113	0.652	0.232	0.163	0.122	0.876	-0.093	0.921
18	C3	-0.042	0.939	0.066	0.687	0.132	0.786	-0.027	0.973
19	CP1	-0.072	0.737	0.032	0.896	0.2	0.559	0.007	0.959
20	C5	0.078	0.738	0.207	0.330	0.129	0.839	-0.043	0.868
21	T3	0.11	0.607	0.071	0.870	0.257	0.431	-0.098	0.975
22	TP7	0.072	0.761	0.177	0.452	0.18	0.721	-0.056	0.929
23	CP5	0.061	0.707	0.177	0.401	0.199	0.694	-0.018	0.985
24	P5	0.07	0.661	0.181	0.330	0.23	0.629	-0.002	0.989
25	P3	0.085	0.577	0.191	0.332	0.126	0.883	-0.092	0.955
26	A1	0.014	0.921	0.076	0.823	0.298	0.37	0.014	0.95
27	T5	0.074	0.694	0.122	0.505	0.182	0.661	-0.006	0.964
28	P1	0.114	0.579	0.15	0.439	0.048	0.921	-0.116	0.878
29	P9	0.123	0.477	0.193	0.287	0.224	0.477	0.044	0.989

30	PO3	0.159	0.450	0.200	0.259	0.037	0.991	-0.100	0.89
31	Pz	0.081	0.864	0.127	0.577	0.09	0.977	-0.101	0.953
32	O1	0.185	0.374	0.214	0.209	0.101	0.851	0.011	0.939
33	POz	0.093	0.717	0.139	0.567	0.034	0.969	-0.144	0.894
34	Oz	0.124	0.673	0.142	0.547	0.071	0.93	-0.089	0.814
35	PO4	0.132	0.617	0.129	0.559	0.024	0.943	-0.139	0.95
36	O2	0.115	0.731	0.193	0.285	0.067	0.977	-0.103	0.897
37	P2	0.174	0.371	0.178	0.413	-0.004	0.975	-0.179	0.676
38	CP2	0.187	0.433	0.215	0.363	0.251	0.418	-0.027	0.964
39	P4	0.200	0.257	0.277	0.082	0.095	0.963	-0.141	0.736
40	P10	-0.015	0.914	0.039	0.873	0.191	0.67	-0.018	0.998
41	T6	0.144	0.472	0.247	0.174	0.233	0.422	0.015	0.917
42	P6	0.101	0.694	0.196	0.273	0.203	0.615	-0.001	0.994
43	CP6	0.186	0.355	0.254	0.154	0.188	0.798	-0.013	0.934
44	A2	-0.027	0.878	0.049	0.844	0.256	0.48	0.079	0.878
45	TP8	0.101	0.748	0.167	0.353	0.166	0.796	0.021	0.912
46	C6	0.136	0.580	0.187	0.432	0.145	0.785	-0.031	0.989
47	C4	0.125	0.670	0.224	0.252	0.159	0.786	-0.047	0.986
48	C2	0.035	0.846	0.09	0.847	0.074	0.848	-0.088	0.839
49	T4	0.068	0.671	0.157	0.462	0.224	0.574	-0.115	0.928
50	FC4	0.199	0.436	0.223	0.300	0.07	0.924	-0.131	0.832
51	FC2	0.093	0.806	0.114	0.748	0.029	0.986	-0.096	0.988
52	FT8	0.160	0.345	0.171	0.337	0.018	0.986	-0.101	0.970
53	FC6	0.211	0.307	0.262	0.095	0.039	0.974	-0.143	0.793
54	F8	0.044	0.907	0.124	0.523	0.172	0.769	-0.123	0.854
55	F6	0.14	0.44	0.194	0.245	0.006	0.99	-0.178	0.829
56	F4	0.045	0.963	0.144	0.446	0.059	0.894	-0.195	0.607
57	Cz	0.119	0.615	0.172	0.396	0.202	0.536	-0.100	0.891

Supplementary Table S21. Correlation analysis result of alpha relative power and HF-HRV.

		Group 1. Depressive disorder				Group 2. Anxiety disorder			
Electrodes		Resting		Task		Resting		Task	
No.	Name	r	p (FDR)	r	p (FDR)	r	p (FDR)	r	p (FDR)
01	AF4	0.249	0.308	0.043	0.951	-0.001	0.998	-0.116	0.845
02	F2	0.240	0.396	0.056	0.906	0.021	0.881	-0.116	0.915
03	FCz	0.159	0.469	0.065	0.848	0.121	0.828	-0.094	0.968
04	Fp2	0.204	0.506	-0.092	0.925	-0.019	0.894	-0.154	0.974
05	Fz	0.167	0.619	-0.065	0.994	-0.023	0.946	-0.157	0.866
06	FC1	0.148	0.647	0.039	0.919	0.125	0.686	-0.092	0.970
07	AFz	0.209	0.394	-0.051	0.898	0.000	0.998	-0.181	0.787
08	F1	0.210	0.503	0.010	0.942	0.000	0.999	-0.140	0.817
09	Fp1	0.192	0.624	-0.104	0.833	-0.001	0.994	-0.183	0.604
10	AF3	0.176	0.622	-0.032	0.884	-0.023	0.908	-0.156	0.808
11	F3	0.145	0.670	0.033	0.925	0.036	0.913	-0.092	0.933
12	F5	0.177	0.497	0.077	0.747	0.019	0.963	-0.109	0.711
13	FC5	0.192	0.507	0.103	0.827	0.080	0.674	-0.092	0.743
14	FC3	0.233	0.393	0.073	0.774	0.131	0.47	-0.140	0.603
15	C1	0.085	0.800	-0.010	0.943	0.189	0.539	-0.067	0.833
16	F7	0.097	0.773	0.063	0.833	0.160	0.778	-0.036	0.86
17	FT7	0.192	0.401	0.123	0.531	0.085	0.753	-0.105	0.821
18	C3	0.109	0.690	-0.048	0.784	0.148	0.564	-0.110	0.933
19	CP1	0.053	0.867	-0.067	0.752	0.183	0.599	-0.062	0.975
20	C5	0.210	0.449	0.030	0.825	0.179	0.498	-0.167	0.773
21	T3	0.106	0.979	0.066	0.804	0.209	0.421	-0.047	0.94
22	TP7	0.243	0.37	0.056	0.766	0.195	0.513	-0.134	0.999
23	CP5	0.228	0.418	0.005	0.969	0.196	0.505	-0.117	0.931
24	P5	0.205	0.534	0.045	0.837	0.166	0.739	-0.076	0.886
25	P3	0.206	0.437	0.061	0.862	0.069	0.93	-0.173	0.985
26	A1	0.152	0.85	0.027	0.985	0.265	0.172	-0.021	0.954
27	T5	0.196	0.611	0.122	0.604	0.169	0.528	-0.047	0.965
28	P1	0.18	0.429	0.105	0.72	0.071	0.879	-0.146	0.802
29	P9	0.205	0.656	0.066	0.816	0.177	0.565	-0.097	0.865

30	PO3	0.196	0.362	0.107	0.806	0.103	0.804	-0.109	0.84
31	Pz	0.153	0.558	0.071	0.917	0.141	0.754	-0.093	0.849
32	O1	0.24	0.378	0.232	0.159	0.103	0.72	-0.119	0.835
33	POz	0.163	0.425	0.088	0.831	0.14	0.7	-0.126	0.899
34	Oz	0.198	0.442	0.192	0.304	0.111	0.719	-0.157	0.773
35	PO4	0.166	0.566	0.108	0.703	0.122	0.75	-0.15	0.799
36	O2	0.205	0.527	0.191	0.300	0.107	0.725	-0.162	0.874
37	P2	0.231	0.46	0.157	0.459	0.086	0.833	-0.158	0.708
38	CP2	0.236	0.349	0.135	0.599	0.264	0.175	-0.063	0.75
39	P4	0.259	0.233	0.16	0.443	0.114	0.732	-0.144	0.775
40	P10	0.097	0.942	-0.03	0.895	0.186	0.522	-0.064	0.916
41	T6	0.155	0.65	0.091	0.937	0.204	0.452	-0.023	0.935
42	P6	0.150	0.778	0.035	0.876	0.21	0.417	-0.062	0.856
43	CP6	0.192	0.629	0.069	0.812	0.176	0.661	-0.103	0.83
44	A2	0.024	0.943	-0.045	0.743	0.248	0.249	0.007	0.963
45	TP8	0.107	0.864	0.002	0.990	0.205	0.452	-0.007	0.974
46	C6	0.188	0.718	-0.034	0.805	0.186	0.485	-0.011	0.937
47	C4	0.217	0.505	0.054	0.900	0.202	0.383	-0.031	0.922
48	C2	0.183	0.56	0.065	0.919	0.175	0.532	-0.064	0.842
49	T4	0.012	0.928	-0.03	0.977	0.128	0.575	-0.019	0.966
50	FC4	0.271	0.332	0.068	0.904	0.149	0.522	-0.083	0.814
51	FC2	0.169	0.554	0.073	0.882	0.163	0.52	-0.03	0.896
52	FT8	0.212	0.302	0.074	0.702	0.001	0.996	-0.088	0.889
53	FC6	0.26	0.284	0.075	0.801	0.043	0.977	-0.105	0.728
54	F8	0.025	0.931	-0.034	0.822	0.102	0.944	-0.011	0.937
55	F6	0.227	0.285	0.071	0.798	0.019	0.988	-0.081	0.879
56	F4	0.111	0.684	-0.014	0.990	0.01	0.995	-0.088	0.933
57	Cz	0.052	0.894	-0.005	0.974	0.18	0.498	-0.059	0.783

Supplementary Table S22. Correlation analysis result of beta absolute power and HF-HRV.

		Group 1. Depressive disorder				Group 2. Anxiety disorder			
Electrodes		Resting		Task		Resting		Task	
No.	Name	r	p (FDR)	r	p (FDR)	r	p (FDR)	r	p (FDR)
01	AF4	-0.155	0.541	0.089	0.654	0.012	0.988	-0.191	0.54
02	F2	-0.124	0.550	0.140	0.467	-0.022	0.874	-0.090	0.821
03	FCz	0.023	0.917	0.134	0.624	0.145	0.645	0.085	0.897
04	Fp2	-0.095	0.786	0.127	0.571	0.025	0.897	-0.132	0.776
05	Fz	-0.072	0.884	0.087	0.708	-0.023	0.997	-0.172	0.562
06	FC1	-0.163	0.756	0.009	0.946	0.180	0.485	0.109	0.863
07	AFz	-0.106	0.627	0.111	0.599	0.025	0.928	-0.114	0.779
08	F1	-0.097	0.934	0.032	0.848	0.085	0.897	-0.056	0.814
09	Fp1	-0.066	0.894	0.038	0.844	0.024	0.864	-0.134	0.736
10	AF3	-0.116	0.933	0.021	0.894	-0.048	0.949	-0.180	0.625
11	F3	-0.092	0.931	0.087	0.757	-0.007	0.963	-0.147	0.853
12	F5	-0.161	0.833	0.042	0.905	0.078	0.916	-0.074	0.804
13	FC5	-0.132	0.733	0.141	0.542	0.108	0.818	-0.040	0.842
14	FC3	-0.2	0.607	0.076	0.809	0.121	0.778	0.064	0.917
15	C1	-0.178	0.612	0.019	0.969	0.152	0.614	0.033	0.969
16	F7	-0.088	0.904	-0.048	0.895	0.164	0.667	-0.117	0.828
17	FT7	-0.192	0.754	0.022	0.894	0.106	0.820	-0.023	0.94
18	C3	-0.247	0.290	-0.038	0.950	0.120	0.86	0.024	0.998
19	CP1	-0.191	0.517	-0.002	0.991	0.167	0.62	0.019	0.939
20	C5	-0.189	0.659	0.015	0.98	0.099	0.937	0.051	0.923
21	T3	0.032	0.841	0.022	0.942	0.176	0.525	-0.087	0.936
22	TP7	-0.204	0.569	-0.049	0.998	0.107	0.853	0.02	0.984
23	CP5	-0.204	0.425	-0.013	0.923	0.115	0.873	0.072	0.907
24	P5	-0.166	0.875	0.015	0.989	0.156	0.759	0.061	0.982
25	P3	-0.177	0.581	0.033	0.975	0.134	0.754	0.04	0.873
26	A1	-0.201	0.446	-0.091	0.858	0.151	0.77	-0.087	0.981
27	T5	-0.194	0.655	-0.118	0.829	0.129	0.707	-0.004	0.977
28	P1	-0.095	0.739	0.039	0.931	0.111	0.674	-0.037	0.968
29	P9	-0.066	0.969	0.051	0.995	0.133	0.731	0.022	0.978

30	PO3	-0.1	0.67	0.11	0.762	0.122	0.638	-0.053	0.845
31	Pz	-0.161	0.648	0.029	0.837	0.164	0.535	-0.073	0.991
32	O1	-0.016	0.98	0.076	0.753	0.168	0.719	0.113	0.769
33	POz	-0.154	0.567	0.026	0.85	0.116	0.687	-0.083	0.996
34	Oz	-0.106	0.973	-0.048	0.917	0.15	0.521	0.038	0.998
35	PO4	-0.087	0.931	0.002	0.989	0.102	0.75	-0.046	0.985
36	O2	-0.115	0.875	0.108	0.796	0.137	0.694	0.01	0.996
37	P2	-0.054	0.85	0.073	0.995	0.1	0.873	-0.036	0.998
38	CP2	-0.02	0.915	0.096	0.815	0.196	0.472	0.023	1
39	P4	-0.074	0.652	0.173	0.492	0.116	0.849	-0.033	0.989
40	P10	-0.166	0.96	-0.02	0.982	0.177	0.67	0.024	0.983
41	T6	-0.07	0.862	0.186	0.451	0.214	0.653	0.068	0.81
42	P6	-0.1	0.96	0.118	0.637	0.19	0.69	0.075	0.982
43	CP6	-0.033	0.99	0.127	0.782	0.168	0.774	0.055	0.962
44	A2	-0.106	0.895	0.000	0.999	0.235	0.361	0.103	0.98
45	TP8	-0.059	0.991	0.082	0.749	0.136	0.852	0.042	0.968
46	C6	-0.039	0.964	0.114	0.798	0.11	0.893	0.001	0.997
47	C4	-0.081	0.907	0.127	0.714	0.132	0.886	0.026	0.922
48	C2	-0.137	0.954	-0.05	0.938	0.107	0.718	-0.005	0.971
49	T4	-0.02	0.954	0.096	0.895	0.194	0.524	-0.095	0.899
50	FC4	-0.041	0.999	0.119	0.627	0.091	0.947	-0.021	0.994
51	FC2	-0.074	0.89	-0.007	0.98	0.07	0.921	-0.016	0.935
52	FT8	-0.111	0.996	0.015	0.928	0.097	0.91	-0.01	0.987
53	FC6	-0.117	0.953	0.13	0.611	0.084	0.849	-0.031	0.972
54	F8	-0.051	0.998	0.092	0.754	0.123	0.816	-0.091	0.964
55	F6	-0.146	0.793	0.032	0.943	0.041	0.907	-0.1	0.977
56	F4	-0.147	0.942	0.083	0.641	0.005	0.987	-0.174	0.712
57	Cz	-0.01	0.943	0.091	0.840	0.193	0.602	-0.064	0.968

Supplementary Table S23. Correlation analysis result of beta relative power and HF-HRV.

		Group 1. Depressive disorder				Group 2. Anxiety disorder			
Electrodes		Resting		Task		Resting		Task	
No.	Name	r	p (FDR)	r	p (FDR)	r	p (FDR)	r	p (FDR)
01	AF4	-0.148	0.647	-0.406	**0.025***	-0.057	0.862	-0.049	0.854
02	F2	-0.158	0.611	-0.423	**0.015***	0.013	0.924	0.014	0.922
03	FCz	-0.152	0.479	-0.286	0.119	0.021	0.96	-0.022	0.924
04	Fp2	-0.148	0.563	-0.346	0.116	-0.092	0.827	0.117	0.703
05	Fz	-0.151	0.703	-0.29	0.247	-0.05	0.849	-0.113	0.77
06	FC1	-0.108	0.620	-0.238	0.265	0.041	0.991	0.047	0.939
07	AFz	-0.218	0.341	-0.46	**0.005***	-0.024	0.903	-0.09	0.762
08	F1	-0.166	0.474	-0.379	**0.026***	0.011	0.952	0.005	0.973
09	Fp1	-0.141	0.655	-0.348	0.064	-0.104	0.730	0.118	0.744
10	AF3	-0.162	0.503	-0.423	**0.010***	-0.068	0.752	-0.066	0.843
11	F3	-0.145	0.648	-0.248	0.214	-0.077	0.754	0.068	0.93
12	F5	-0.175	0.42	-0.342	0.072	-0.071	0.865	0.006	0.968
13	FC5	-0.1800	0.399	-0.324	0.097	-0.044	0.753	0.024	0.942
14	FC3	-0.193	0.334	-0.334	0.051	0.000	0.998	0.006	0.966
15	C1	-0.124	0.526	-0.245	0.178	0.043	0.962	0.041	0.909
16	F7	-0.209	0.277	-0.313	0.162	-0.069	0.763	0.091	0.834
17	FT7	-0.208	0.252	-0.326	0.125	-0.076	0.873	0.013	0.972
18	C3	-0.182	0.263	-0.313	**0.049***	0.028	0.969	0.034	0.972
19	CP1	-0.140	0.550	-0.233	0.218	0.064	0.789	0.007	0.961
20	C5	-0.227	0.171	-0.351	**0.028***	0.024	0.983	0.040	0.985
21	T3	-0.182	0.417	-0.14	0.624	0.001	0.993	-0.054	0.828
22	TP7	-0.23	0.164	-0.373	**0.028***	-0.041	0.831	0.010	0.961
23	CP5	-0.229	0.165	-0.36	**0.021***	-0.028	0.961	0.043	0.935
24	P5	-0.207	0.205	-0.373	**0.018***	-0.082	0.74	-0.002	0.987
25	P3	-0.209	0.255	-0.324	**0.048***	-0.014	0.971	0.085	0.963
26	A1	-0.201	0.225	-0.328	**0.046***	-0.119	0.953	-0.095	0.891
27	T5	-0.18	0.343	-0.322	0.067	-0.117	0.844	-0.065	0.863
28	P1	-0.201	0.255	-0.292	0.080	0.011	0.939	0.054	0.966
29	P9	-0.197	0.272	-0.33	0.054	-0.099	0.778	-0.116	0.893

30	PO3	-0.219	0.218	-0.327	0.057	0.02	0.908	0	0.998
31	Pz	-0.254	0.185	-0.337	0.053	0.046	0.876	0.04	0.971
32	O1	-0.237	0.17	-0.349	**0.025***	-0.047	0.844	0	0.998
33	POz	-0.22	0.225	-0.361	**0.035***	0.001	0.992	-0.023	0.95
34	Oz	-0.206	0.265	-0.306	0.056	-0.07	0.837	-0.043	0.939
35	PO4	-0.237	0.229	-0.342	**0.032***	0.011	0.941	-0.02	0.925
36	O2	-0.218	0.231	-0.312	0.050	-0.056	0.868	-0.068	0.979
37	P2	-0.223	0.199	-0.332	**0.032***	0.008	0.952	0.035	0.955
38	CP2	-0.181	0.375	-0.314	0.052	-0.012	0.934	-0.039	0.957
39	P4	-0.162	0.434	-0.324	**0.041***	-0.006	0.965	0.004	0.977
40	P10	-0.062	0.903	-0.252	0.160	-0.066	0.814	-0.029	0.955
41	T6	-0.103	0.731	-0.298	0.121	-0.113	0.901	-0.026	0.977
42	P6	-0.096	0.776	-0.3	0.065	-0.041	0.918	-0.042	0.883
43	CP6	-0.12	0.618	-0.277	0.102	-0.022	0.948	-0.02	0.988
44	A2	-0.034	0.915	-0.245	0.191	-0.139	0.961	-0.082	0.944
45	TP8	-0.072	0.866	-0.216	0.287	-0.037	0.897	-0.027	0.923
46	C6	-0.088	0.751	-0.241	0.177	-0.028	0.841	-0.003	0.984
47	C4	-0.087	0.786	-0.213	0.330	-0.007	0.962	0.047	0.983
48	C2	-0.092	0.694	-0.246	0.291	0.003	0.985	-0.026	0.902
49	T4	-0.186	0.499	-0.31	0.085	-0.217	0.375	0.002	0.986
50	FC4	-0.095	0.738	-0.237	0.243	0.028	0.97	0.026	0.956
51	FC2	-0.089	0.666	-0.205	0.336	0.013	0.924	-0.032	0.82
52	FT8	-0.142	0.5	-0.227	0.325	-0.032	0.901	0.005	0.973
53	FC6	-0.142	0.558	-0.302	0.154	-0.003	0.982	0.037	0.974
54	F8	-0.222	0.187	-0.281	0.330	-0.09	0.862	0.164	0.63
55	F6	-0.122	0.602	-0.297	0.171	-0.043	0.887	0.022	0.966
56	F4	-0.15	0.638	-0.245	0.531	-0.079	0.784	-0.004	0.975
57	Cz	-0.145	0.466	-0.266	0.123	0.013	0.926	-0.032	0.965

Supplementary Table S24. Correlation analysis result of gamma absolute power and HF-HRV.

Electrodes		Group 1. Depressive disorder				Group 2. Anxiety disorder			
		Resting		Task		Resting		Task	
No.	Name	r	p (FDR)	r	p (FDR)	r	p (FDR)	r	p (FDR)
01	AF4	-0.132	0.481	0.245	0.180	-0.073	0.804	-0.231	0.318
02	F2	-0.115	0.575	0.275	0.169	-0.133	0.592	-0.127	0.599
03	FCz	0.053	0.776	0.169	0.544	0.072	0.783	0.059	0.885
04	Fp2	-0.086	0.766	0.167	0.441	-0.015	0.97	-0.135	0.787
05	Fz	-0.048	0.981	0.118	0.505	-0.033	0.813	-0.128	0.787
06	FC1	-0.153	0.421	0.108	0.868	0.144	0.664	0.083	0.867
07	AFz	-0.08	0.804	0.240	0.244	-0.002	0.987	-0.093	0.656
08	F1	-0.048	0.858	0.102	0.843	0.067	0.868	-0.138	0.594
09	Fp1	0.046	0.858	0.047	0.791	-0.022	0.889	-0.149	0.76
10	AF3	-0.022	0.970	0.134	0.528	-0.13	0.898	-0.235	0.38
11	F3	-0.045	0.858	0.112	0.741	-0.072	0.978	-0.15	0.785
12	F5	-0.103	0.649	0.085	0.794	0.057	0.92	-0.161	0.523
13	FC5	-0.065	0.749	0.190	0.393	0.075	0.956	-0.172	0.596
14	FC3	-0.138	0.448	0.214	0.365	0.04	0.976	0.085	0.903
15	C1	-0.129	0.497	0.105	0.883	0.13	0.615	-0.016	0.94
16	F7	-0.073	0.891	0.002	0.991	0.091	0.919	-0.141	0.839
17	FT7	-0.14	0.438	0.081	0.804	0.048	0.938	-0.097	0.725
18	C3	-0.185	0.314	0.062	0.880	-0.013	0.923	-0.019	0.922
19	CP1	-0.146	0.409	0.146	0.525	0.037	0.965	-0.05	0.963
20	C5	-0.144	0.506	0.083	0.748	-0.112	0.683	0.042	0.999
21	T3	0.009	0.984	0.006	0.967	0.075	0.963	-0.129	0.908
22	TP7	-0.185	0.45	-0.026	0.991	-0.066	0.829	-0.007	0.972
23	CP5	-0.168	0.465	0.076	0.755	-0.06	0.864	0.085	0.842
24	P5	-0.155	0.42	0.078	0.771	0.051	0.942	-0.028	0.982
25	P3	-0.143	0.383	0.121	0.539	0.067	0.925	-0.002	0.989
26	A1	-0.214	0.294	-0.098	0.881	0.058	0.949	-0.219	0.393
27	T5	-0.223	0.213	-0.247	0.49	0.039	0.846	-0.084	0.849
28	P1	-0.084	0.634	0.097	0.927	0.079	0.954	-0.102	0.938
29	P9	-0.117	0.593	0.01	0.944	0.112	0.782	-0.023	0.999

30	PO3	-0.115	0.575	0.184	0.571	0.075	0.763	-0.098	0.865
31	Pz	-0.127	0.506	0.06	0.798	0.036	0.862	-0.111	0.836
32	O1	-0.054	0.751	-0.069	0.845	0.259	0.521	0.061	0.91
33	POz	-0.134	0.471	0.029	0.903	0.044	0.89	-0.11	0.929
34	Oz	-0.134	0.422	-0.207	0.693	0.15	0.613	0.072	0.971
35	PO4	-0.077	0.746	-0.019	0.997	-0.002	0.99	-0.101	0.859
36	O2	-0.129	0.638	0.1	0.69	0.107	0.636	0.018	0.953
37	P2	-0.083	0.642	0.096	0.858	-0.008	0.964	-0.088	0.875
38	CP2	-0.045	0.803	0.152	0.78	0.208	0.553	0.113	0.999
39	P4	-0.096	0.568	0.154	0.595	0.017	0.949	-0.113	0.978
40	P10	-0.134	0.468	-0.123	0.859	0.044	0.925	-0.144	0.632
41	T6	-0.045	0.744	0.182	0.431	0.096	0.906	-0.066	0.796
42	P6	-0.127	0.469	0.147	0.752	0.124	0.971	-0.050	0.914
43	CP6	0.000	1.000	0.131	0.73	0.051	0.928	-0.067	0.985
44	A2	0.016	0.908	0.048	0.908	0.083	0.999	-0.017	0.932
45	TP8	-0.013	0.938	0.141	0.653	-0.036	0.961	-0.087	0.867
46	C6	-0.024	0.859	0.269	0.239	-0.033	0.946	-0.143	0.934
47	C4	-0.07	0.656	0.215	0.422	-0.019	0.928	-0.075	0.975
48	C2	-0.169	0.306	-0.056	0.928	0.065	0.87	0.001	0.995
49	T4	0.106	0.827	0.129	0.795	0.173	0.678	-0.166	0.829
50	FC4	-0.033	0.81	0.242	0.266	0.01	0.969	-0.098	0.954
51	FC2	-0.032	0.885	-0.01	0.978	-0.034	0.948	-0.028	0.955
52	FT8	-0.094	0.578	0.054	0.886	-0.01	0.942	-0.107	0.679
53	FC6	-0.083	0.588	0.251	0.424	-0.025	0.856	-0.109	0.862
54	F8	-0.04	0.778	0.151	0.737	0.048	0.874	-0.133	0.913
55	F6	-0.121	0.514	0.124	0.675	-0.066	0.831	-0.154	0.602
56	F4	-0.094	0.712	0.196	0.345	-0.024	0.916	-0.145	0.811
57	Cz	-0.009	0.95	0.112	0.826	0.131	0.842	-0.104	0.829

Supplementary Table S25. Correlation analysis result of gamma relative power and HF-HRV.

		Group 1. Depressive disorder				Group 2. Anxiety disorder			
Electrodes		Resting		Task		Resting		Task	
No.	Name	r	p (FDR)	r	p (FDR)	r	p (FDR)	r	p (FDR)
01	AF4	-0.132	0.440	-0.142	0.551	-0.061	0.932	-0.053	0.992
02	F2	-0.133	0.511	-0.164	0.424	-0.002	0.988	0.097	0.916
03	FCz	-0.106	0.474	-0.170	0.404	0.018	0.994	0.097	0.837
04	Fp2	-0.105	0.701	-0.160	0.848	-0.028	0.992	-0.025	0.884
05	Fz	-0.068	0.835	-0.207	0.528	0.046	0.915	0.094	0.945
06	FC1	-0.093	0.535	-0.096	0.626	-0.009	0.949	0.082	0.962
07	AFz	-0.088	0.677	-0.209	0.387	0.013	0.928	0.011	0.999
08	F1	-0.091	0.719	-0.224	0.181	0.007	0.999	-0.011	0.996
09	Fp1	-0.054	0.941	-0.249	0.430	-0.05	0.906	-0.083	0.991
10	AF3	-0.039	0.913	-0.195	0.295	-0.064	0.934	-0.097	0.956
11	F3	-0.107	0.704	-0.174	0.472	-0.047	0.852	-0.049	0.855
12	F5	-0.095	0.633	-0.147	0.455	-0.059	0.998	-0.044	0.987
13	FC5	-0.092	0.566	-0.122	0.544	-0.06	0.990	-0.031	0.876
14	FC3	-0.108	0.606	-0.097	0.621	-0.082	0.972	0.098	0.901
15	C1	-0.072	0.706	-0.103	0.534	-0.026	0.967	0.083	0.848
16	F7	-0.141	0.713	-0.199	0.460	-0.214	0.417	-0.053	0.706
17	FT7	-0.151	0.401	-0.187	0.312	-0.091	0.887	0.025	0.921
18	C3	-0.106	0.57	-0.11	0.540	-0.07	0.735	0.105	0.953
19	CP1	-0.081	0.674	-0.087	0.620	-0.086	0.87	0.067	0.914
20	C5	-0.159	0.33	-0.149	0.442	-0.131	0.763	0.128	0.982
21	T3	-0.153	0.659	-0.202	0.35	-0.071	0.993	-0.152	0.549
22	TP7	-0.208	0.271	-0.185	0.279	-0.131	0.925	0.124	0.788
23	CP5	-0.167	0.388	-0.146	0.457	-0.133	0.876	0.144	0.909
24	P5	-0.165	0.426	-0.219	0.214	-0.125	0.819	0.072	0.897
25	P3	-0.127	0.456	-0.155	0.329	-0.045	0.973	0.128	0.986
26	A1	-0.223	0.256	-0.212	0.217	-0.13	0.802	-0.127	0.666
27	T5	-0.207	0.400	-0.292	0.094	-0.124	0.785	0.014	0.98
28	P1	-0.113	0.53	-0.168	0.311	0.009	0.993	0.039	0.956
29	P9	-0.233	0.219	-0.259	0.117	-0.054	0.848	0.016	0.984

30	PO3	-0.147	0.365	-0.204	0.244	0.035	0.949	0.027	0.918
31	Pz	-0.13	0.435	-0.198	0.525	-0.071	0.887	-0.012	0.931
32	O1	-0.157	0.544	-0.311	0.085	0.114	0.692	0.087	0.805
33	POz	-0.134	0.448	-0.21	0.312	-0.039	0.902	0.057	0.962
34	Oz	-0.151	0.579	-0.273	0.136	0.012	0.931	0.139	0.538
35	PO4	-0.114	0.477	-0.195	0.326	-0.062	0.887	0.069	0.725
36	O2	-0.146	0.568	-0.178	0.386	0.009	0.959	0.109	0.604
37	P2	-0.154	0.479	-0.183	0.293	-0.048	0.996	0.082	0.766
38	CP2	-0.173	0.457	-0.174	0.288	-0.018	0.993	0.133	0.901
39	P4	-0.13	0.443	-0.191	0.256	-0.088	0.937	0.069	0.841
40	P10	-0.051	0.84	-0.223	0.255	-0.145	0.503	-0.063	0.727
41	T6	-0.064	0.754	-0.149	0.357	-0.184	0.395	-0.035	0.803
42	P6	-0.102	0.589	-0.169	0.293	-0.109	0.8	0.004	0.977
43	CP6	-0.074	0.696	-0.198	0.267	-0.087	0.971	0	0.999
44	A2	0.04	0.785	-0.112	0.534	-0.181	0.468	-0.019	0.894
45	TP8	-0.078	0.661	-0.111	0.474	-0.116	0.854	-0.012	0.986
46	C6	-0.117	0.479	-0.063	0.707	-0.095	0.839	-0.028	0.913
47	C4	-0.131	0.484	-0.111	0.542	-0.079	0.829	0.081	0.979
48	C2	-0.132	0.541	-0.185	0.280	-0.016	0.911	0.093	0.98
49	T4	-0.065	0.632	-0.149	0.467	-0.146	0.897	-0.162	0.852
50	FC4	-0.127	0.459	-0.112	0.537	-0.034	0.876	0.071	0.997
51	FC2	-0.076	0.684	-0.157	0.369	-0.02	0.956	0.086	0.997
52	FT8	-0.145	0.372	-0.124	0.627	-0.069	0.81	0.021	0.981
53	FC6	-0.135	0.466	-0.129	0.498	-0.053	0.911	0.053	0.991
54	F8	-0.127	0.539	-0.146	0.768	-0.193	0.36	-0.053	0.765
55	F6	-0.14	0.565	-0.114	0.681	-0.072	0.918	0.029	0.987
56	F4	-0.055	0.746	-0.139	0.619	-0.076	0.797	0.052	0.765
57	Cz	-0.062	0.703	-0.217	0.200	0.002	0.986	0.004	0.98
